# Legumain Restrains Granuloma Formation by Inhibiting mTORC1/STAT1‐Mediated M1 Macrophage Polarization in Sarcoidosis

**DOI:** 10.1002/advs.202520635

**Published:** 2026-04-03

**Authors:** Mengyuan Liu, Yueyin Han, Bingbing Xie, Lili Zhu, Wenxiu Xu, Yinzhen Han, Yue Liao, Shuwei Gao, Dingyuan Jiang, Jing Geng, Zhen Li, Yinan Hu, Huaping Dai

**Affiliations:** ^1^ China‐Japan Friendship Hospital (Institute of Clinical Medical Sciences) Chinese Academy of Medical Sciences & Peking Union Medical College Beijing China; ^2^ National Center for Respiratory Medicine State Key Laboratory of Respiratory Health and Multimorbidity National Clinical Research Center for Respiratory Diseases Institute of Respiratory Medicine Chinese Academy of Medical Sciences & Peking Union Medical College Department of Pulmonary and Critical Care Medicine Center of Respiratory Medicine China‐Japan Friendship Hospital Beijing China; ^3^ Immune Dysfunction and Pulmonary Fibrosis Joint Laboratory for Clinical Medicine Capital Medical University Beijing China; ^4^ Department of Pulmonary and Critical Care Medicine Beijing Hospital National Center of Gerontology Institute of Geriatric Medicine Chinese Academy of Medical Science Beijing China

**Keywords:** granulomas, legumain, lipid nanoparticles, macrophages, sarcoidosis

## Abstract

Sarcoidosis is a systemic granulomatous disease that has limited treatment options. Emerging evidence suggests that macrophages are essential for sarcoid granuloma initiation. Legumain (LGMN), a cysteine protease, regulates macrophage polarization in various cancers. However, its involvement in sarcoid granuloma formation remains elusive. Herein, LGMN is upregulated in macrophages within sarcoid‐like granulomas. Genetic deletion of *Lgmn* exacerbates granulomatous inflammation in a *Propionibacterium acnes* (*P. acnes*)‐induced mouse model, accompanied by increased M1 macrophage polarization. Mechanistically, LGMN binds to integrin αvβ3 on the macrophage surface and restrains M1 polarization by inhibiting the mechanistic target of rapamycin complex 1 (mTORC1)/signal transducer and activator of transcription 1 (STAT1) pathway. Furthermore, intratracheal administration of lipid nanoparticles carrying *Lgmn* plasmid DNA effectively alleviates granuloma formation induced by *P. acnes* or trehalose 6,6'‐dimycolate, concomitant with decreased mTORC1/STAT1 activation and M1 polarization. These findings reveal the pivotal role of LGMN in restraining sarcoid granulomatous inflammation through suppression of mTORC1/STAT1‐driven M1 macrophage polarization. Therefore, LGMN supplementation may be a promising therapeutic strategy for sarcoidosis.

## Graphical abstract

Legumain (LGMN) is upregulated in macrophages during sarcoid‑like granuloma formation. Macrophage‑derived LGMN binds to integrin αvβ3 and suppresses mTORC1/STAT1 signaling to restrain M1 macrophage polarization. Intratracheal delivery of lipid nanoparticles carrying *Lgmn* plasmid DNA (pDNA) elevates LGMN expression and effectively attenuates pulmonary granulomatous inflammation.

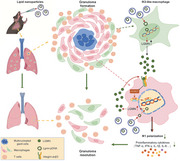

## 1 Introduction

Sarcoidosis is a systemic granulomatous disease of unknown etiology that is pathologically characterized by the presence of non‐caseating epithelioid granulomas predominantly affecting the lungs and intrathoracic lymph nodes [1, 2]. Although approximately two‐thirds of the patients with sarcoidosis achieve clinical remission within several years, the remaining one‐third may exhibit chronic progression, leading to irreversible fibrocystic architectural distortion of the lungs, which is associated with respiratory failure and increased mortality [2, 3]. Despite extensive research on alternative therapeutic approaches, prednisone remains the recommended first‐line treatment for both pulmonary and extrapulmonary sarcoidosis [4]. Nevertheless, prolonged glucocorticoid administration is often limited by significant toxicity, and many patients experience disease relapse during dose tapering [5]. Therefore, there is an urgent need to elucidate the molecular mechanisms underlying granuloma formation and identify novel therapeutic targets for sarcoidosis.

Sarcoid granulomas exhibit a distinct immunological profile characterized by activated macrophages and highly polarized Th1 and Th17 cells. This pattern aligns with persistent antigen stimulation and the concomitant secretion of proinflammatory cytokines [6]. Macrophages constitute the structural core of sarcoid granulomas and play a pivotal role in their initiation [7, 8]. Functionally, macrophages are categorized into two distinct activation states: classically activated (M1) and alternatively activated (M2). Previous studies have shown a correlation between M1 and M2 polarization and sarcoidosis. Chronic upregulation of the mechanistic target of rapamycin complex 1 (mTORC1) signaling pathway in macrophages has been established as a critical regulator of sarcoid‐like granuloma persistence, leading to enhanced glycolysis and pentose–phosphate pathway activation [8, 9, 10, 11], which represents the metabolic signature of M1‐polarized macrophages [12]. An elevated number of CD40^+^ M1 macrophages has also been observed in the airspaces of patients with pulmonary sarcoidosis [13]. However, both a SodA‐induced granulomatous mouse model and an in vitro granuloma model exhibited significant upregulation of M2 polarization markers [14, 15]. Thus, targeting macrophage polarization may represent a potential therapeutic strategy for sarcoidosis; however, the predominant polarization phenotype during sarcoid granuloma formation remains controversial and warrants further investigation.

Legumain (LGMN), also called asparagine endopeptidase, is a member of the C13 family of cysteine proteases that hydrolyzes the C‐terminus of asparagine residues [16, 17]. Despite its predominant localization in endolysosomal compartments, where it mediates physiological protein catabolism [18], LGMN can exert its functions in the cytoplasm, on cell surfaces, and in extracellular environments [19, 20]. LGMN is highly overexpressed in a range of pathological conditions, including cancer, neurodegenerative disorders, cardiovascular diseases, organ fibrosis, and inflammatory diseases [19, 20, 21]. In tumor tissues, LGMN is mainly derived from tumor‐associated macrophages (TAMs) [22, 23]. In thoracic aortic dissection and renal fibrosis, LGMN co‐localizes with the M2 marker CD206, and its level is significantly elevated in interleukin (IL)‐4‐induced M2 macrophages [20, 24]. Previous studies have demonstrated that LGMN promotes M2 polarization and facilitates M1‐to‐M2 macrophage transition [22, 25, 26]. Although these findings suggest that LGMN influences macrophage polarization, its specific role in modulating macrophage dynamics and granuloma formation in sarcoidosis remains unclear.

In this study, we observed significant upregulation of LGMN in macrophages during sarcoid‐like granuloma formation. Additionally, we found that macrophage‐derived LGMN suppressed mTORC1/STAT1signaling by binding to integrin αvβ3 on the macrophage surface, thereby inhibiting M1 polarization. Genetic deletion of *Lgmn* in mice exacerbated *Propionibacterium acnes* (*P. acnes*)‐induced sarcoid‐like granuloma formation. Notably, the overexpression of *Lgmn* via lipid nanoparticles (LNPs) loaded with *Lgmn* plasmid DNA (pDNA) mitigated granulomatous inflammation in *P. acnes*‐and trehalose 6,6'‐dimycolate (TDM)‐administered mice, highlighting the therapeutic potential of LGMN in sarcoidosis.

## 2 Results

### 2.1 LGMN is Upregulated in Human and Mouse Granulomatous Tissues

**FIGURE 1:**
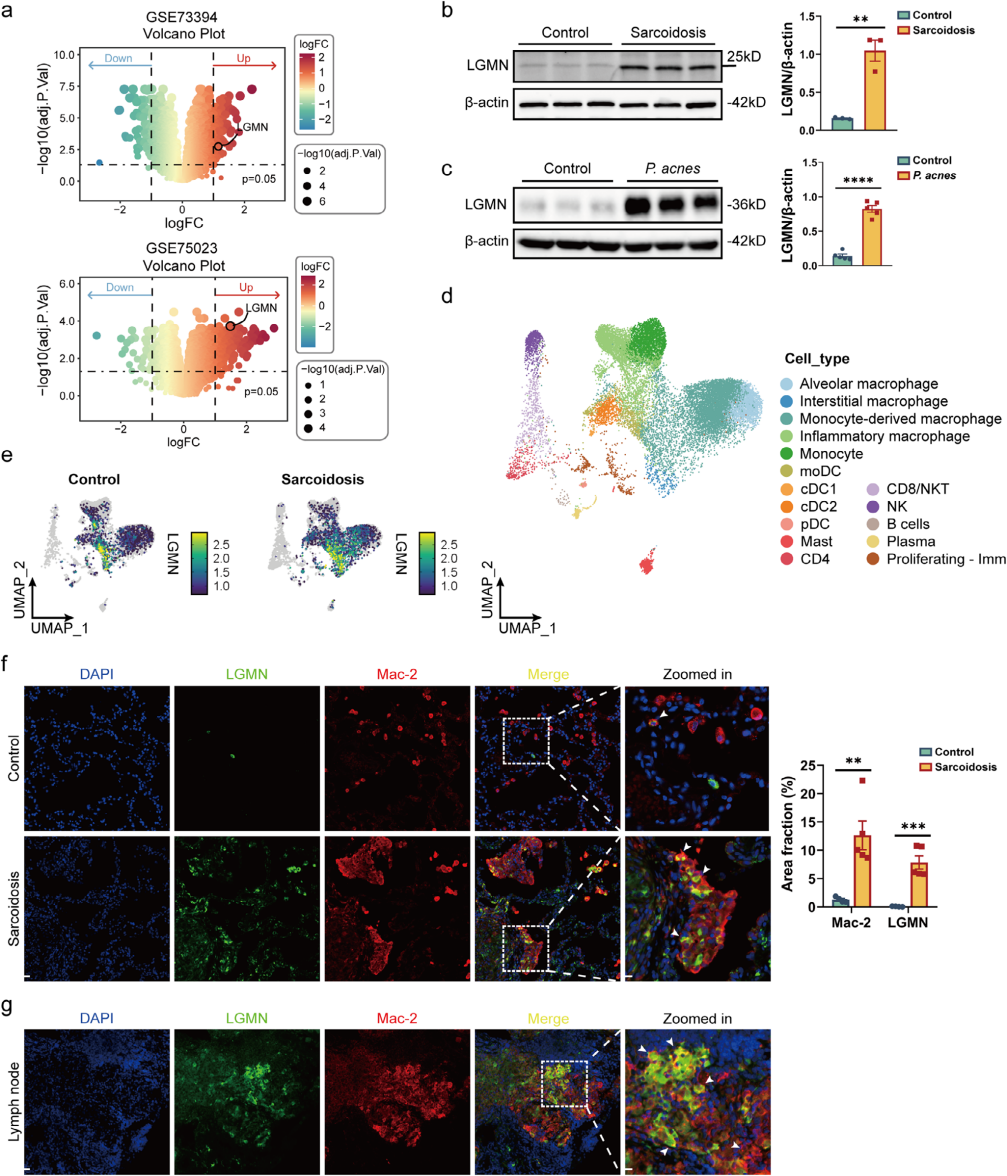
LGMN is upregulated in human and mouse granulomatous tissues. (a) Volcano plot of DEGs in the BAL cells of patients with sarcoidosis compared to those of healthy donors. Transcriptomic data sources: GSE73394, GSE75023. (b) Western blot analysis and quantification for LGMN expression in the lymph nodes of control subjects and sarcoidosis patients (n = 3 each). The β‐actin blot shown in this panel is shared with Figure 3a, and all bands in both panels were derived from the same original membrane. Full‐length blots are provided in Supporting Information Figure S11. (c) Western blot analysis and quantification for LGMN expression in the lungs of PBS (Control) and *P. acnes‐*induced mouse model (n  =  5 each). (d) UMAP of scRNA‐seq profiles from lungs of sarcoidosis patients (Sarcoidosis) (n = 4) and donors (Control) (n = 5). Transcriptomic data sources: GSE227136. (e) UMAP from (d) annotated with expression of LGMN. (f) Results for co‐immunostaining of LGMN (green) and the macrophage marker Mac‐2 (red) in the lung sections from patients with sarcoidosis (n = 5) and control subjects (n = 4). The nuclei were stained blue by DAPI. The images were acquired at an original magnification of ×400 (Bar = 20 µm), with a corresponding zoomed‑in view shown at higher magnification (Bar = 10 µm). (g) Representative images for co‐immunostaining for LGMN (green) and Mac‐2 (red) in the lymph node sections from patients with sarcoidosis (n = 5). The nuclei were stained blue by DAPI. The images were captured at an original magnification of ×400 (Bar = 20 µm), and a zoomed‑in inset of the indicated region is shown at higher magnification (Bar = 10 µm). The data are represented as the mean ± SEM. An unpaired *t*‐test was applied. * *p* < 0.05, ** *p* < 0.01, *** *p* < 0.001, **** *p* < 0.0001.

To investigate LGMN expression levels in patients with pulmonary sarcoidosis, we retrieved bulk RNA sequencing data from two GEO datasets (GSE73394 and GSE75023). Both datasets contained transcriptomic profiles derived from bronchoalveolar lavage (BAL) cells. Differential expression analysis was performed using the limma package in R following standard normalization and statistical procedures. When applying a significance threshold of adjusted *p* < 0.05 and |log_2_ fold change| > 1, 29 upregulated genes were identified. These included inflammation‐associated genes, particularly cytokines and chemokines (CCL18, CXCL10, and CCL5), and T cell biomarkers (CD2, CD3E, and TRAC). Among the differentially expressed genes (DEGs), LGMN was significantly upregulated in the BAL cells of patients with pulmonary sarcoidosis (Figure 1a; Figure S1a, Supporting Information). To validate the increased expression of LGMN protein, we examined its levels in the lymph nodes of patients with sarcoidosis and in the lungs of mice with *P. acnes*‐induced granulomas. Consistent with our transcriptomic findings, LGMN protein expression was markedly elevated in the lymph nodes of patients with sarcoidosis compared with those of healthy controls (Figure 1b). Similarly, the lung homogenates of mice following *P. acnes* induction exhibited significantly higher LGMN levels than those of control mice (Figure 1c).

To determine the cellular source and localization of LGMN within sarcoid granulomas, we analyzed single‐cell RNA sequencing (scRNA‐seq) data from lung tissues of patients with sarcoidosis and healthy donors retrieved from the GEO series (GSE227136). Using annotations established from the published dataset, we identified 16 distinct cell clusters (Figure 1d). We visualized LGMN expression across the identified cell clusters using FeaturePlot, revealing elevated LGMN levels in the sarcoidosis group compared with those in the healthy control group. Notably, LGMN was primarily expressed in macrophages, with predominant localization in monocyte‐derived and interstitial macrophages (Figure 1e). Similarly, analysis of the granulomatous murine skin scRNA‐seq dataset (GSE250508) demonstrated marked upregulation of *Lgmn* mRNA levels in the granulomatous skin of *Tsc2* ^fl/fl^ CD11c‐Cre^+^ sarcoidosis mice (*Tsc2*‐cKO) compared with those in the normal skin of *Tsc2* ^fl/fl^ controls (Figure S1b,c, Supporting Information). Co‐immunostaining showed that LGMN was nearly undetectable in lung sections from control subjects, whereas sarcoidosis patient‐derived lung tissues exhibited prominent aggregates of macrophage with high levels of LGMN (Figure 1f). Additionally, consistent with the bioinformatics findings, we confirmed that LGMN was primarily localized in macrophages within granulomas of both the lungs and lymph nodes from patients with sarcoidosis (Figure 1f,g), as well as in pulmonary granulomas of *P. acnes*‐administered mice (Figure S1d, Supporting Information). These findings suggest that LGMN expression is elevated in sarcoidosis granulomas, with macrophages being the principal cellular source.

Given the high levels of LGMN within sarcoidosis granulomas and its secretory nature, we next evaluated its diagnostic potential. We measured LGMN concentrations in bronchoalveolar lavage fluid (BALF) from patients with sarcoidosis to assess their correlation with disease activity, estimated by the BALF CD4/CD8 ratio and serum angiotensin‑converting enzyme (ACE) levels. Notably, LGMN levels in BALF did not differ significantly between patients with low versus high CD4/CD8 ratios or serum ACE levels (Figure S2a,b, Supporting Information). Because protein concentrations in BALF can be affected by sampling variability, and to further evaluate the diagnostic value of LGMN in pulmonary sarcoidosis, we reanalyzed the GSE109516 dataset containing BAL cell transcriptomic profiles. As described in the Experimental Section, patients were stratified by the presence of pulmonary parenchymal involvement according to their Scadding stage. In this dataset, LGMN mRNA levels were significantly higher in patients with pulmonary parenchymal involvement (Figure S2c,d, Supporting Information). A threshold of 99.63 transcripts per million (TPM) was identified using the Youden index and subsequently validated by internal cross‑validation (Figure S2e, Supporting Information). To assess the robustness and generalizability of this threshold, we further validated it in the external dataset GSE73394. In this dataset, a percentile‑matched threshold of 98.78 TPM yielded an AUC of 0.815 (95% CI: 0.676‐0.955), with a sensitivity of 76.9% and a specificity of 80.0% (Figure S2f–h, Supporting Information), supporting the stability of LGMN as a diagnostic marker.

### 2.2 *Lgmn* Deficiency Exacerbates Lung Granulomatous Inflammation

**FIGURE 2:**
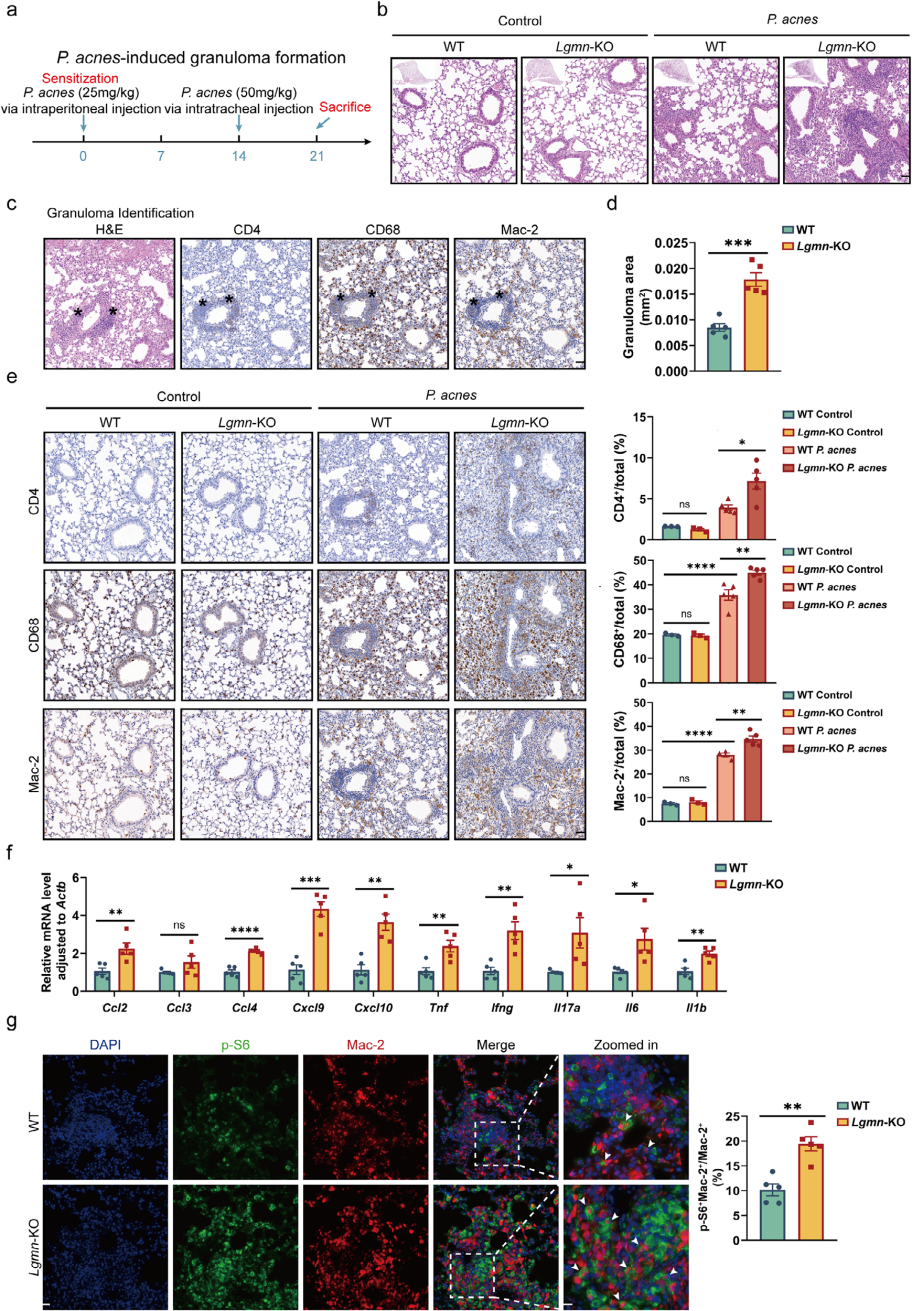
*Lgmn* deficiency exacerbates lung granulomatous inflammation. (a) Schematic of *P. acnes* induction in WT and *Lgmn*‐KO mice. (b) Representative images of hematoxylin and eosin (H&E)‐stained lung sections from the mice described in (a). Images were captured at ×200 magnification. Bar = 50 µm. (c) H&E staining (left) and immunohistochemistry (IHC) for CD4, CD68, and Mac‐2 (right) showing granuloma‐like areas in the lungs of *P. acnes‐*challenged mice. Asterisks (*) indicate granulomas. Bar = 50 µm. (d) A bar graph showed the mean granuloma area of *P. acnes‐*elicited lung granulomas in WT and *Lgmn‐*KO mice (*n*  =  4‐5 each). (e) IHC staining and quantification for the levels of CD4 and macrophage markers (CD68 and Mac‐2) in the lung sections from control (n = 3 per genotype) and *P. acnes*‐induced WT and *Lgmn*‐KO mice (n = 5 per genotype). Representative images were captured at ×200 magnification. Bar = 50 µm. (f) qRT‐PCR analysis of *Ccl2, Ccl3, Ccl4, Cxcl9, Cxcl10, Tnf, Ifng, Il17a, Il6*, and *Il1b* expression in the lung homogenates from WT and *Lgmn‐*KO mice after *P. acnes* induction (n = 5 each). (g) Results for co‐immunostaining of p‐S6 (green) and Mac‐2 (red) in the lung sections from WT and *Lgmn‐*KO mice after *P. acnes* induction (n = 5 each). The nuclei were stained blue by DAPI. Representative images were captured at an original magnification of ×400 (Bar = 20 µm), with a zoomed‑in view of the indicated region shown at higher magnification (Bar = 10 µm). The data are represented as the mean ± SEM. An unpaired *t*‐test (d, f, g) and ordinary one‐way ANOVA (e) were applied. * *p* < 0.05, ** *p* < 0.01, *** *p* < 0.001, **** *p* < 0.0001.

To elucidate the role of LGMN in sarcoid‐like granulomatous inflammation, we generated *Lgmn‐*KO mice and assessed their susceptibility to *P. acnes*‐induced granuloma formation (Figure 2a). All mice were genotyped with PCR (Figure S3a, Supporting Information). The absence of *Lgmn* was validated by quantitative real‐time polymerase chain reaction (qRT‐PCR) (Figure S3b, Supporting Information). Representative photomicrographs of pulmonary granulomas induced by *P. acnes* in WT and *Lgmn*‐KO mice are shown in Figure 2b. Granuloma‑like areas were identified according to the morphological features illustrated in Figure 2c and were quantified thereafter. H&E staining revealed larger and more compact epithelioid granulomas localized to the bronchoarterial bundle in *Lgmn*‐KO mice on day 21 following *P. acnes* sensitization, and the mean granuloma area was significantly larger in *Lgmn*‐KO mice than in WT mice (Figure 2b,d).

We then examined the extent of pulmonary inflammation. IHC analysis demonstrated an increased proportion of CD4‐positive T cells, CD68‐positive macrophages, and Mac‐2‐positive macrophages in the lungs of *Lgmn*‐KO mice exposed to *P. acnes*, indicating enhanced infiltration of the principal effector cells involved in granuloma formation, particularly macrophages and CD4^+^ T cells (Figure 2e). Additionally, we quantified the mRNA expression levels of inflammatory cytokines and chemokines previously reported in sarcoidosis using qRT‐PCR. Elevated mRNA levels of chemokines (*Ccl2, Ccl4, Cxcl9*, and *Cxcl10*) and inflammatory cytokines (*Tnf, Ifng, Il17a*, *Il6*, and *Il1b*) were observed in the lungs of *Lgmn*‐KO mice compared to those of WT mice after *P. acnes* induction, suggesting that *Lgmn* deficiency enhances the production of granuloma‐associated cytokines (Figure 2f). Given the pivotal role of mTORC1 signaling in macrophages during granuloma formation, we evaluated the phosphorylation levels of the S6 ribosomal protein (p‐S6). Immunofluorescence staining revealed elevated levels of p‐S6 within the granulomas of *Lgmn*‐KO mice, accompanied by an increased number of p‐S6‐positive macrophages in the lungs, compared with those in WT mice (Figure 2g; Figure S4, Supporting Information).

Taken together, our findings demonstrate that *Lgmn* deficiency exacerbates pulmonary granulomatous inflammation induced by *P. acnes*.

### 2.3 *Lgmn* Deficiency Switches Macrophages Toward M1 Polarization

**FIGURE 3:**
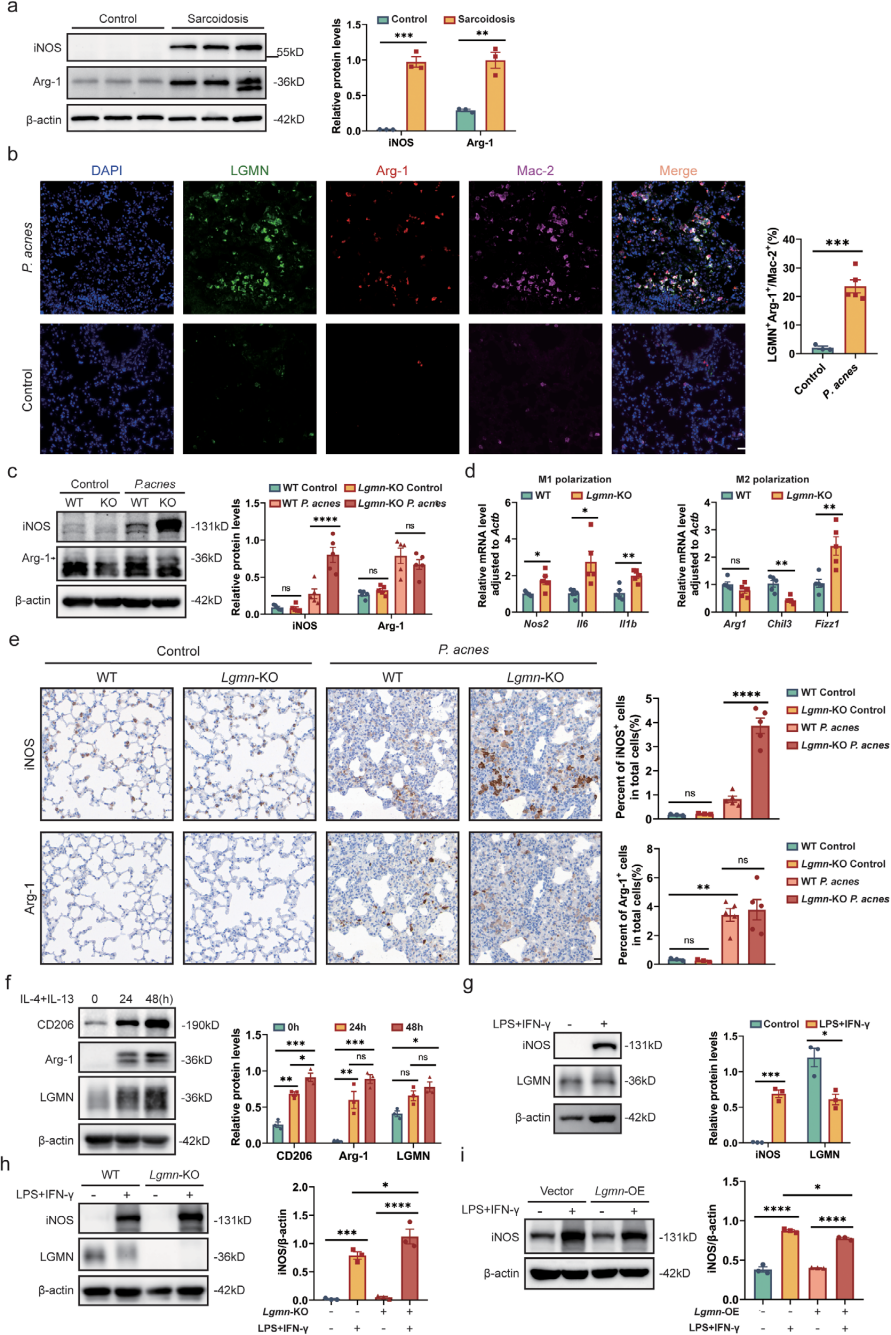
*Lgmn* deficiency switches macrophages toward M1 polarization. (a) Western blot analysis of protein levels of iNOS and Arg‐1 in the lymph nodes of control subjects and sarcoidosis patients (n = 3 each). The β‐actin blot in this panel is shared with Figure 1b, and all bands were derived from the same membrane. Full‐length blots are provided in Figure S11 of the Supporting Information. (b) Representative graphs for co‐immunostaining of LGMN (green), Arg‐1 (an M2 polarization marker) (red), and Mac‐2 (purple) in the lung sections from Control (n = 3) and *P. acnes*‐induced mice (n = 5). The nuclei were stained blue by DAPI. Images were captured at ×400 magnification. Bar = 20 µm. (c) Western blot analysis of iNOS and Arg‐1 expression in the lungs from WT and *Lgmn*‐KO mice after *P. acnes* challenge (n = 5 each). (d) qRT‐PCR analysis of M1 polarization markers (*Nos2, Il6*, and *Il1b*) *and* M2 polarization markers (*Arg1, Chil3*, and *Fizz1*) expression in the lung homogenates from WT and *Lgmn‐*KO mice after *P. acnes* induction (n = 5 each). (e) IHC staining and quantification of iNOS and Arg‑1 expression in lung sections from control WT and *Lgmn*‑KO mice (n = 3 each) and *P. acnes*–induced WT and *Lgmn*‑KO mice (n = 5 each). Images were captured at ×400 magnification. Bar = 20 µm. (f) Western blot analysis of the levels of CD206, Arg‐1, and LGMN in bone marrow‐derived macrophages (BMDMs) following stimulation with IL‐4 and IL‐13. (g) Western blot analysis of iNOS and LGMN expression in BMDMs 12 h after stimulation with LPS and IFN‐γ. (h) Western blot analysis of iNOS and LGMN expression in WT and *Lgmn*‐KO BMDMs following stimulation with LPS and IFN‐γ. (i) Western blot analysis of the levels of iNOS in vector‐ or *Lgmn* plasmid (*Lgmn*‐OE)‐treated RAW264.7 cells 12 h after LPS and IFN‐γ induction. The data are represented as the mean ± SEM. An unpaired *t*‐test (a, b, d, g) and ordinary one‐way ANOVA (c, e, f, h, i) were applied. * *p* < 0.05, ** *p* < 0.01, *** *p* < 0.001, **** *p* < 0.0001.

Previous studies have highlighted the crucial role of macrophage polarization in granuloma formation in patients with sarcoidosis [15, 27]. To investigate this further, we analyzed the phenotypic profiles of macrophages in the lymph nodes of patients with sarcoidosis and healthy controls. Western blot analysis revealed markedly elevated expression of both M1 (iNOS) and M2 (Arg‐1) markers in sarcoidosis lymph nodes compared to that in control lymph nodes (Figure 3a). Consistent results were observed in the lungs of the WT mice exposed to *P. acnes*, which exhibited upregulated mRNA and protein levels of both M1 and M2 markers (Figure S5a,b, Supporting Information). These findings suggest simultaneous activation of M1 and M2 macrophage polarization in sarcoid granulomas. Co‐immunostaining for LGMN, Arg‐1, and Mac‐2 demonstrated that LGMN was predominantly localized within M2‐like macrophages in the granulomatous regions (Figure 3b). To delineate the specific role of LGMN in macrophage polarization during sarcoid‐like granuloma formation, we assessed the expression of macrophage polarization markers in the lungs of *P. acnes*‐induced WT and *Lgmn*‐KO mice. Western blotting and qRT‐PCR analyses revealed that *Lgmn* deletion significantly enhanced both mRNA synthesis and protein expression of M1 markers, whereas M2 marker expression showed inconsistent alterations (Figure 3c,d). Similar patterns were observed in the lung sections by IHC staining for iNOS and Arg‐1(Figure 3e). Collectively, these results indicate that *Lgmn* deficiency promotes a shift toward M1 macrophage polarization while exerting limited effects on M2 polarization in vivo.

We further validated the effect of LGMN on macrophage polarization in vitro. Consistently, LGMN protein expression was significantly upregulated following stimulation with IL‐4 and IL‐13 for 48 h, whereas its expression was markedly downregulated upon stimulation with lipopolysaccharide (LPS) and interferon‐γ (IFN‐γ) (Figure 3f,g), suggesting preferential expression of LGMN in M2‐like macrophages. To evaluate the functional role of LGMN in macrophages in vitro, BMDMs were isolated from WT and *Lgmn*‐KO mice and subsequently treated with LPS and IFN‐γ. Western blot analysis revealed elevated iNOS protein levels in *Lgmn*‐KO BMDMs compared to those in their WT counterparts following stimulation (Figure 3h). Additionally, *Lgmn* plasmid was employed to enhance LGMN expression, which was confirmed by Western blotting in HEK‐293T cells (Figure S5c, Supporting Information). Similarly, LGMN overexpression in RAW264.7 cells via plasmid transfection significantly suppressed iNOS protein expression induced by LPS and IFN‐γ (Figure 3i).

Overall, these results indicate that *Lgmn* deficiency, particularly in macrophages, may promote M1 macrophage polarization, thereby enhancing sarcoid‐like granulomatous inflammation.

### 2.4 LGMN–Integrin αvβ3 Interaction Suppresses STAT1‐Mediated M1 Polarization

**FIGURE 4:**
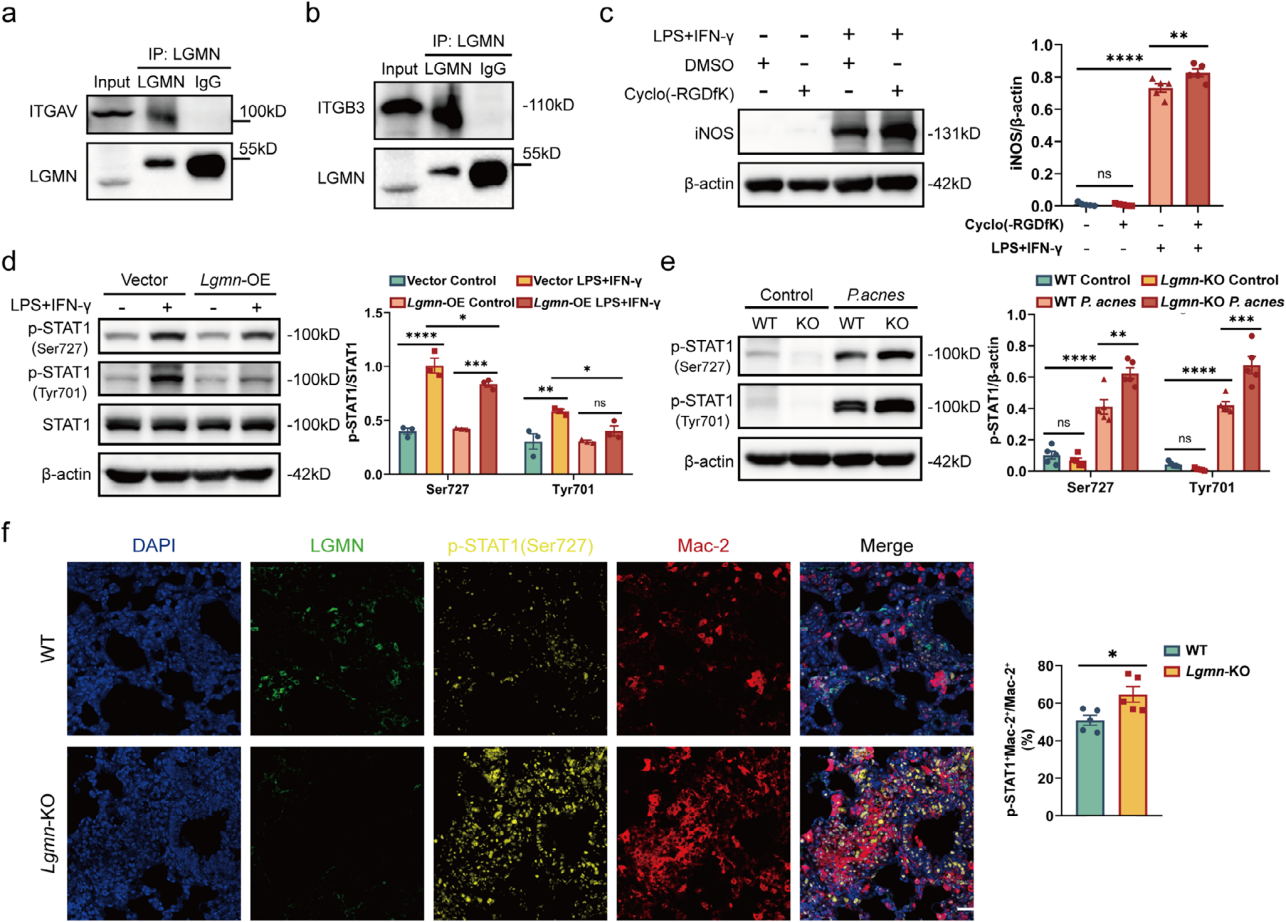
LGMN–integrin αvβ3 interaction suppresses STAT1‐mediated M1 polarization. (a) Co‐immunoprecipitation of ITGAV and LGMN in the lung homogenate from the *P. acnes*‐induced mouse model. (b) Co‐immunoprecipitation of ITGB3 and LGMN in the lung homogenate from *the P. acnes*‐induced mouse model. (c) Western blot analysis of iNOS expression in BMDMs after stimulation with LPS and IFN‐γ with or without integrin αvβ3 antagonist Cyclo(‐RGDfK). (d) Western blot analysis of the levels of p‐STAT1 (Ser727 and Tyr701) and STAT1 in vector‐ or *Lgmn* plasmid‐treated RAW264.7 cells stimulated with LPS and IFN‐γ. (e) Western blot analysis of p‐STAT1 (Ser727 and Tyr701) expression in the lungs from WT and *Lgmn*‐KO mice after *P. acnes* challenge (n = 5 each). (f) Results for co‐immunostaining of LGMN (green), p‐STAT1 (Ser727) (yellow), and Mac‐2 (red) in the lung sections from WT and *Lgmn‐*KO mice after *P. acnes* challenge (n = 5 each). The nuclei were stained blue by DAPI. Representative images were captured at ×400 magnification. Bar = 25 µm. The data are represented as the mean ± SEM. An unpaired *t*‐test (f) and ordinary one‐way ANOVA (c, d, e) were applied. * *p* < 0.05, ** *p *< 0.01, *** *p* < 0.001, **** *p* < 0.0001.

Integrin αvβ3, a heterodimer consisting of the αv (ITGAV) and β3 (ITGB3) subunits, has been identified as the receptor for LGMN in multiple cell types, including vascular smooth muscle cells and TAMs [19, 20, 23]. To investigate whether LGMN interacts with integrin αvβ3 in sarcoid‐like granulomas, lung tissues from *P. acnes*‐induced mice were subjected to co‐immunoprecipitation. Using anti‐LGMN antibodies, we immunoprecipitated ITGAV and ITGB3 and observed that both subunits were co‐immunoprecipitated with LGMN (Figure 4a,b), supporting a physical interaction between LGMN and integrin αvβ3. To elucidate the role of LGMN–integrin αvβ3 interaction in M1 polarization, BMDMs were treated with Cyclo(‐RGDfK)—a selective antagonist that blocks integrin αvβ3 binding with its ligands. This treatment upregulated iNOS protein levels in BMDMs upon stimulation of LPS and IFN‐γ (Figure 4c), suggesting that LGMN negatively regulates M1 polarization via its interaction with integrin αvβ3.

LGMN has been reported to suppress STAT1 activation by interacting with integrin αvβ3 in TAMs [23]. Given the pivotal role of JAK1/STAT1 signaling in M1 polarization [28], we evaluated the effect of LGMN on STAT1 activation in vitro. Western blot analysis revealed decreased phosphorylation of STAT1 at both Ser727 and Tyr701 in RAW264.7 cells transfected with *Lgmn* plasmid, whereas total STAT1 protein levels remained unchanged (Figure 4d). We further evaluated whether LGMN inhibited M1 macrophage programming via JAK1/STAT1 signaling during granuloma formation in vivo. We detected JAK1 and STAT1 phosphorylation in the WT and *Lgmn*‐KO mice. Compared with WT mice, elevated expression of phosphorylated STAT1 (p‐STAT1) at both Ser727 and Tyr701 was observed in the lungs of *Lgmn*‐KO mice exposed to *P. acnes*, whereas no statistically significant differences were detected in p‐JAK1 protein levels between the WT and *Lgmn*‐KO mice (Figure 4e; Figure S6a,b, Supporting Information). Immunofluorescence staining consistently revealed an increased proportion of p‐STAT1(Ser727)‐positive macrophages within the granulomas of *Lgmn*‐KO mice (Figure 4f).

In summary, these results demonstrate that LGMN binds to integrin αvβ3 on the surface of macrophages, thereby inhibiting STAT1 phosphorylation, suppressing M1 macrophage polarization, and ultimately mitigating *P. acnes*‐induced granulomatous inflammation.

### 2.5 LGMN Restrains mTORC1/STAT1 Signaling to Limit M1 Polarization

**FIGURE 5:**
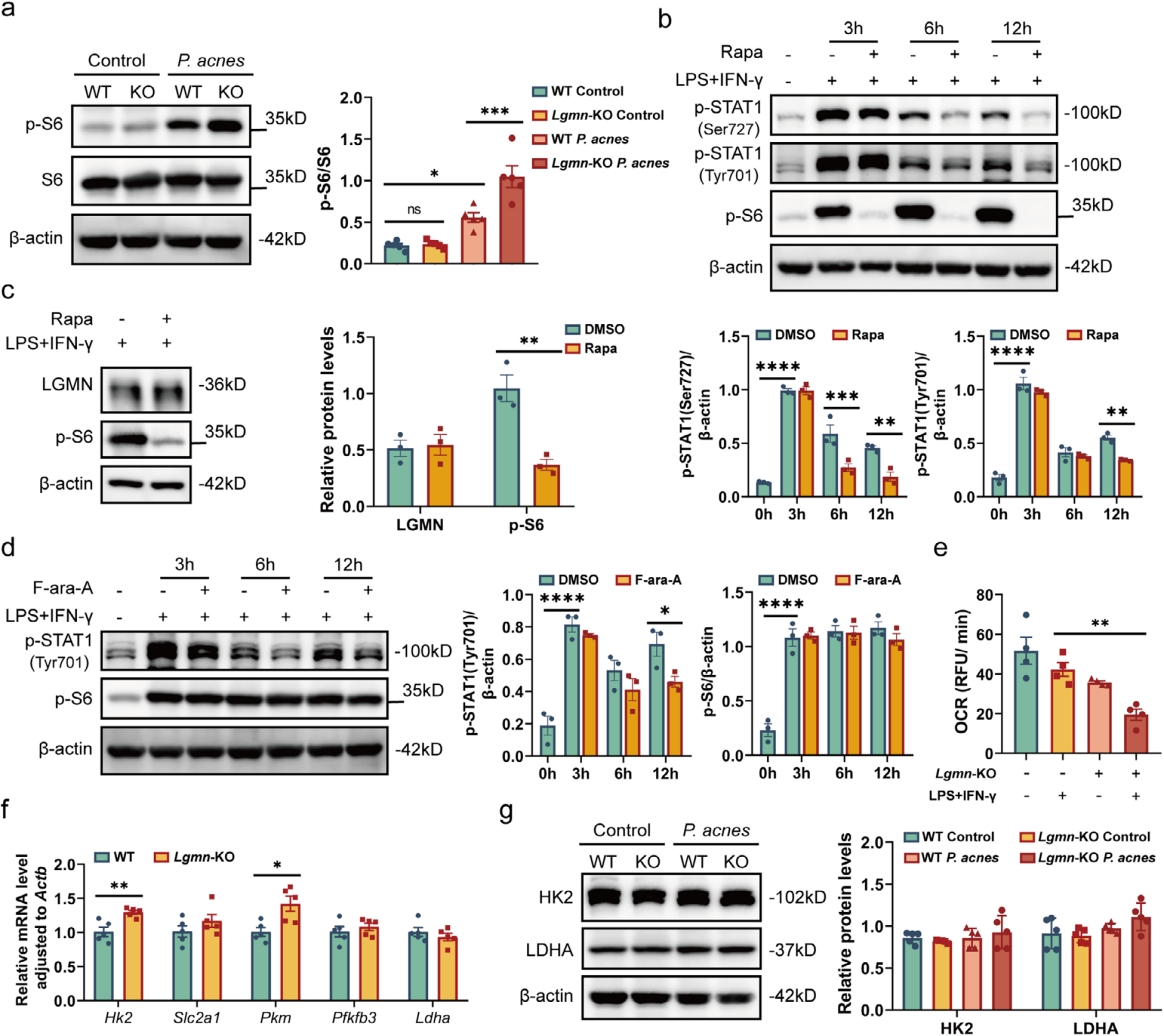
LGMN restrains mTORC1/STAT1 signaling to limit M1 polarization. (a) Western blot analysis of p‐S6 and S6 expression in the lungs from WT and *Lgmn*‐KO mice after *P. acnes* challenge (n = 5 each). (b) Western blot analysis of p‐STAT1 (Ser727 and Tyr701) and p‐S6 expression in BMDMs treated with Rapa for 0, 3, 6, and 12 h. (c) Western blot analysis of the levels of LGMN and p‐S6 in BMDMs treated with or without Rapa for 6 h. (d) Western blot analysis of p‐STAT1(Tyr701) and p‐S6 expression in BMDMs treated with the STAT1 inhibitor F‐ara‐A for 0, 3, 6, and 12 h. (e) OCR was measured in WT or *Lgmn*‐KO BMDMs following stimulation with LPS and IFN‐γ for 12 h (n = 4). (f) qRT‐PCR analysis of glycolysis‐related genes (*Hk2, Slc2a1, Pkm, Pfkfb3*, and *Ldha*) expression in lung homogenates from WT and *Lgmn‐*KO mice after *P. acnes* induction (n = 5 each). (g) Western blot analysis of HK2 and LDHA expression in lung tissues from WT and *Lgmn*‐KO mice after *P. acnes* challenge (n = 5 each). The data are represented as the mean ± SEM. An unpaired *t*‐test (c, f) and ordinary one‐way ANOVA (a, b, d, e, g) were applied. * *p* < 0.05, ** *p* < 0.01, *** *p* < 0.001, **** *p* < 0.0001.

Given the critical role of mTORC1 signaling in sarcoid granuloma formation, we examined whether LGMN regulates macrophage function through this pathway. As described above, co‐immunostaining showed that *Lgmn* deficiency resulted in increased infiltration of p‐S6‐positive macrophages within granulomatous lesions. To quantitatively assess mTORC1 activation, we measured the p‑S6/S6 ratio in the lungs of WT and *Lgmn*‑KO mice challenged by *P. acnes*. Consistently, *Lgmn*‐KO mice exhibited markedly elevated p‐S6/S6 levels compared with WT mice, indicating that *Lgmn* deficiency enhances mTORC1 activation in vivo (Figure 5a).

Having shown that LGMN inhibits both mTORC1 activation and STAT1‐mediated M1 polarization, we next sought to define the regulatory relationship among LGMN, mTORC1, and STAT1. To this end, we treated BMDMs with the mTORC1‐specific inhibitor rapamycin (Rapa) together with LPS and IFN‐γ, and detected STAT1 phosphorylation at multiple time points. Notably, Rapa treatment substantially reduced STAT1 phosphorylation at Ser727 at 6 h, and at both Ser727 and Tyr701 at 12 h following stimulation with LPS and IFN‐γ, indicating that mTORC1 signaling regulates STAT1 phosphorylation in macrophages (Figure 5b). In parallel, LGMN protein levels remained unchanged following Rapa treatment, suggesting that mTORC1 does not feedback to regulate LGMN expression (Figure 5c). To determine whether STAT1 activation reciprocally affects mTORC1 signaling, we stimulated BMDMs with LPS and IFN‐γ alone or in combination with the STAT1 inhibitor fludarabine (F‐ara‐A). Although F‐ara‐A effectively suppressed STAT1 phosphorylation at Tyr701, it had minimal effect on p‐S6 levels, demonstrating that STAT1 does not modulate mTORC1 pathway activity (Figure 5d).

Given that mTORC1 regulates cellular metabolism and growth, and that enhanced glycolysis supports macrophage proliferation during granuloma formation [14], we further explored whether LGMN influences glycolytic reprogramming in macrophages under inflammatory conditions. We first assessed the glycolytic function of *Lgmn*‐deficient macrophages in vitro. Real‐time metabolic analysis showed that upon LPS and IFN‐γ stimulation, *Lgmn*‐KO BMDMs exhibited a further reduction in oxygen consumption rate (OCR) compared with WT BMDMs (Figure 5e), indicating suppressed mitochondrial oxidative phosphorylation. To determine whether this alteration was associated with changes in glycolysis, we examined glycolysis‑related gene expression in the lungs of WT and *Lgmn*‑KO mice. qRT‐PCR analysis revealed significantly higher mRNA levels of key glycolytic enzymes, including *Hk2* and *Pkm*, in *Lgmn*‑KO mice, whereas other glycolytic genes showed no significant differences (Figure 5f). Consistently, Western blotting showed a modest upward trend in HK2 and LDHA protein expression in the lungs of *P. acnes*–challenged *Lgmn*‑KO mice, although the differences were not statistically significant (Figure 5g). These findings suggest a potential alteration in glycolytic metabolism in vivo.

Collectively, these results demonstrate that *Lgmn* deficiency induces mTORC1 activation, which in turn promotes STAT1 phosphorylation and subsequent M1 macrophage polarization. *Lgmn* deficiency is also associated with suppressed oxidative phosphorylation and a modest tendency toward increased glycolytic gene and protein expression in vivo.

### 2.6 Administration of LNPs Carrying *Lgmn* pDNA Protected Mice From Lung Granulomatous Inflammation

**FIGURE 6:**
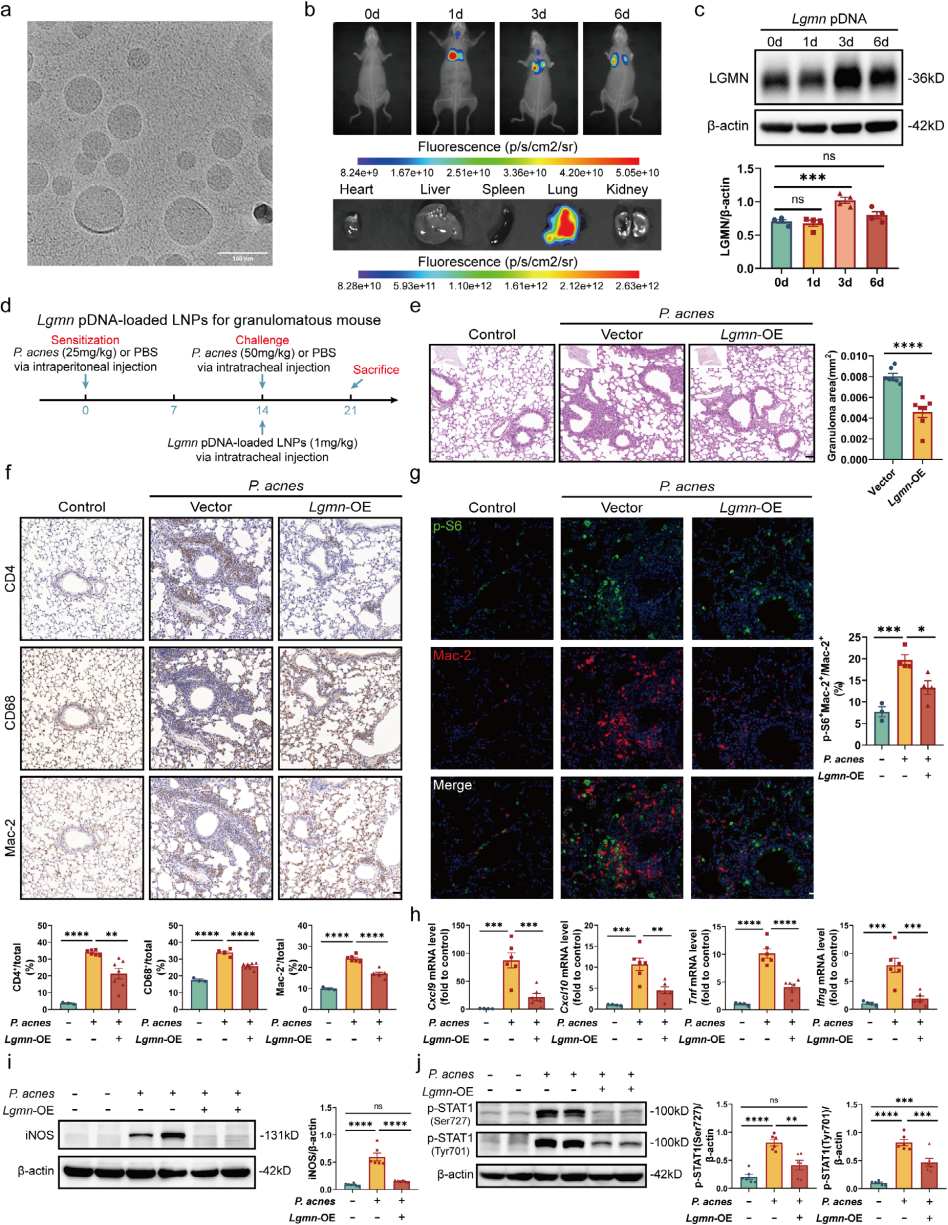
Administration of LNPs carrying *Lgmn* pDNA protected mice from lung granulomatous inflammation. (a) Representative image of *Lgmn* pDNA‐loaded LNPs taken by TEM. Bar = 100 nm. (b) The fluorescence images of mice at different timepoints (n = 4 each) after DiR‐labeled *Lgmn* pDNA‐loaded LNPs intratracheal instillation and the fluorescence intensity of major organs on day 3. (c) Temporal LGMN expression changes in lungs from mice following intratracheal delivery of *Lgmn* pDNA‐loaded LNPs (n = 4 each). (d) Schematic of *Lgmn* pDNA‐loaded LNPs treatment design. (e) Histological analysis of the severity of lung granulomatous inflammation in mice after *P. acnes* induction with or without *Lgmn* pDNA‐loaded LNPs treatment (Control group n = 3, Vector‐loaded LNPs (Vector) group n = 7, *Lgmn* pDNA‐loaded LNPs (*Lgmn*‐OE) group n = 7). Images were captured at ×200 magnification. Bar = 50 µm. (f) IHC staining and quantification for the levels of CD4, CD68, and Mac‐2 in the lung sections from *P. acnes*‐induced mice with or without *Lgmn* pDNA‐loaded LNPs treatment (Control n = 3, Vector n = 6, *Lgmn*‐OE n = 7). Representative images were captured at ×200 magnification. Bar = 50 µm. (g) Results for co‐immunostaining of p‐S6 (green) and Mac‐2 (red) in the lung sections from Control group (n = 3), Vector group (n = 4), and *Lgmn*‐OE group (n = 4). The nuclei were stained blue by DAPI. Images were captured at ×400 magnification. Bar = 20 µm. (h) qRT‐PCR analysis of *Cxcl9, Cxcl10, Tnf*, and *Ifng* expression in the lung homogenates from Control, vector‐loaded LNPs‐, or *Lgmn* pDNA‐loaded LNPs‐ treated mice (n = 4, 6, and 6, respectively). (i) Western blot analysis of iNOS expression in the lungs of Control, vector‐loaded LNPs‐, or *Lgmn* pDNA‐loaded LNPs‐ treated mice (n = 6 each). (j) Western blot analysis of the levels of p‐STAT1 (Ser727 and Tyr701) in the lungs from *P. acnes*‐induced mice with or without *Lgmn* pDNA‐loaded LNPs treatment (n = 6 each). The data are represented as the mean ± SEM. Unpaired *t*‐test (e) and ordinary one‐way ANOVA (c, f, g, h, i, j) were applied. * *p* < 0.05, ** *p* < 0.01, *** *p* < 0.001, **** *p* < 0.0001.

To further validate the protective role of LGMN in sarcoid‐like granulomas and to facilitate the translation of our findings into clinical applications, we developed LNPs loaded with *Lgmn* pDNA. The prepared LNPs demonstrated a 69.15% entrapment efficiency for loading pDNA with a zeta potential of −2.78 mV (Figure S7a, Supporting Information). The *Lgmn* pDNA‐loaded LNPs had a regular spherical morphology with a mean diameter of approximately 100 nm, as observed by transmission electron microscopy (TEM) (Figure 6a). Additionally, the size analysis revealed a normal hydrodynamic diameter distribution of the LNPs, with a mean diameter of 94.11 nm (Figure S7b, Supporting Information). Furthermore, we assessed the mean size of the prepared LNPs at different time points (0, 1, 3, and 6 days), and no obvious change in LNP size was observed within 3 days, indicating the short‐term stability of the LNPs (Figure S7c, Supporting Information).

DiR‐labeled LNPs were prepared and intratracheally administered to mice to determine their biodistribution. After intratracheal administration, the mice were imaged using an in vivo imaging system (IVIS) on days 0, 1, 3, and 6. The fluorescent signal of the LNPs was predominantly localized in the lungs and gradually declined over time. The mice were sacrificed on day 3, and the major organs were harvested for ex vivo fluorescence signal evaluation. Consistently, fluorescent LNPs were mainly distributed in the lungs, and minimal fluorescence signals were detected in other organs (Figure 6b). We further evaluated the potential toxicity of the LNPs in mice using H&E staining. No histopathological lesions were found in the major organs of the mice on days 1, 3, and 6 after the intratracheal injection (Figure S7d, Supporting Information). Serum biochemical parameters of liver and kidney function, together with urinalysis, further supported the biocompatibility and biosafety of *Lgmn* pDNA‐loaded LNPs (Figure S7e, Table S3, Supporting Information). To investigate the temporal dynamics of LGMN expression following LNP administration, we measured LGMN protein levels in the lungs by Western blotting. Marked LGMN upregulation was observed 3 d after intratracheal administration of *Lgmn* pDNA‐loaded LNPs, and LGMN expression returned to baseline on day 6 (Figure 6c).

To assess the therapeutic effects of *Lgmn* pDNA‐loaded LNPs on *P. acnes*‐induced pulmonary granulomas, WT mice were intratracheally administered either an empty vector or *Lgmn* pDNA‐loaded LNPs 14 d after *P. acnes* injection (Figure 6d). H&E staining revealed that *P. acnes*‐induced pulmonary granulomas were attenuated following treatment with *Lgmn* pDNA‐loaded LNPs, with a significant reduction in the mean granuloma area observed in the treatment group (Figure 6e). The reduced infiltration of CD4^+^ T cells and macrophages was confirmed by IHC staining for CD4, CD68, and Mac‐2 (Figure 6f). Co‐immunofluorescence staining for p‐S6 and Mac‐2 demonstrated a marked decrease in the proportion of p‐S6‐positive macrophages in the treatment group, suggesting the suppression of mTORC1 signaling (Figure 6g). Consistently, qRT‐PCR analysis showed a pronounced downregulation of proinflammatory cytokine mRNA levels (*Cxcl9, Cxcl10, Tnf*, and *Ifng*) in mice treated with *Lgmn* pDNA‐loaded LNPs (Figure 6h). These results support the therapeutic efficacy of *Lgmn* pDNA‐loaded LNPs in alleviating sarcoid‐like granulomas.

We further analyzed the polarization phenotype of the macrophages and the downstream signaling pathways of LGMN. Reduced protein levels of iNOS were observed in the treated mice (Figure 6i). Additionally, treatment with *Lgmn* pDNA‐loaded LNPs suppressed the expression of p‐STAT1 at Ser727 and Tyr701 in mice (Figure 6j). Consistently, the proportion of p‐STAT1(Ser727)‐positive macrophages within granulomas significantly decreased in the treatment group (Figure S8, Supporting Information). Our data demonstrated that *Lgmn* pDNA‐loaded LNP administration effectively upregulated LGMN expression, thereby attenuating *P. acnes*‐induced granulomatous inflammation, highlighting its therapeutic potential in sarcoidosis.

To evaluate the therapeutic potential of LGMN overexpression in established granulomas, we first confirmed the formation of granuloma‐like structures on day 17 after *P. acnes* sensitization by H&E staining (Figure S9a, Supporting Information). WT mice were then administered empty vector or *Lgmn* pDNA‐loaded LNPs intratracheally on the same day (Figure S9b). H&E and IHC staining showed that *Lgmn* pDNA‐loaded LNPs exerted significant therapeutic effects even when administered after granuloma formation (Figure S9c–e). *Lgmn* pDNA–loaded LNPs also reduced the elevated p‑S6 levels and p‐STAT1 expression at Tyr701 and Ser727 (Figure S9f). Consistently, qRT‑PCR analysis revealed decreased mRNA levels of proinflammatory cytokines, including *Ccl4, Cxcl9, Cxcl10, Tnf*, and *Ifng* (Figure S9g). Collectively, these findings indicate that airway delivery of *Lgmn* pDNA–loaded LNPs confers therapeutic benefits against established sarcoid‑like granulomas.

To further assess the generalizability of these findings, we employed a TDM‐induced lung granuloma model. H&E staining confirmed pulmonary granuloma formation on day 7 after TDM injection (Figure S10a, Supporting Information). Western blotting showed increased LGMN expression in TDM‑treated lungs, accompanied by elevated iNOS and Arg‑1 levels, indicating the coexistence of M1 and M2 polarization in this model (Figure S10b, Supporting Information). Higher levels of p‑S6 and p‑STAT1 (Ser727 and Tyr701) were also observed in TDM‑injected mice (Figure S10c, Supporting Information). *Lgmn* pDNA‐loaded LNPs also exerted marked therapeutic effects in this model (Figure S10d–g, Supporting Information), significantly reducing proinflammatory cytokine expression (*Ccl4, Cxcl9, Cxcl10, Tnf, Ifng*, and *Il1b*) (Figure S10h, Supporting Information). Consistently, *Lgmn* pDNA‑loaded LNP treatment attenuated M1 polarization markers at both the protein and mRNA levels (Figure S10i,j, Supporting Information) and markedly diminished TDM‑induced p‐STAT1 (Ser727 and Tyr701) and p‑S6 expression (Figure S10k, Supporting Information).

Overall, these findings suggest that *Lgmn* pDNA‐loaded LNP administration effectively upregulated LGMN expression, thereby attenuating *P. acnes*‐ and TDM‐induced granulomatous inflammation, highlighting its therapeutic potential in sarcoidosis.

## 3 Discussion

In this study, experiments were conducted using human biopsy samples and murine models to elucidate the role of LGMN in the pathogenesis of sarcoidosis. We observed elevated LGMN expression in macrophages within the granulomatous lesions from patients with sarcoidosis and *P. acnes*‐induced mouse models. Notably, transcriptomic analysis of BAL cells revealed the diagnostic potential of LGMN mRNA levels for pulmonary involvement in sarcoidosis. Using *Lgmn*‐KO mice, we found that *Lgmn* deficiency exacerbated granulomatous inflammation and was associated with enhanced M1 macrophage polarization. Mechanistically, LGMN interacted with integrin αvβ3 and restrained mTORC1/STAT1 signaling, thereby limiting M1 macrophage polarization and the associated glycolytic reprogramming. Importantly, intratracheal delivery of *Lgmn* pDNA‐loaded LNPs upregulated LGMN expression in mice, leading to the alleviation of sarcoid‐like granuloma formation induced by *P. acnes*. Consistent therapeutic effects were observed in the TDM‑induced granuloma model. Collectively, our study revealed the pivotal role of LGMN–integrin αvβ3 interaction in restraining mTORC1/STAT1‐driven M1 macrophage polarization, consequently attenuating sarcoid granulomatous inflammation. By this means, LGMN overexpression represents a promising strategy for treating sarcoidosis.

Proteases, including the matrix metalloproteinase (MMP) family and cysteine proteases (e.g., Cathepsin K), have been implicated in the pathogenesis of sarcoidosis [29, 30, 31]. Clinical studies have demonstrated increased levels of MMP‐1, ‐2, ‐7, ‐8, ‐9, and ‐12 in the peripheral blood, BALF, and affected tissues of patients with sarcoidosis, which correlate with disease severity [32, 33, 34, 35, 36], LGMN is a cysteine protease primarily localized in the lysosome, where it plays a crucial role in proteolytic processing. It can also be secreted extracellularly, whereby it exerts proteolytic activity or interacts with cell surface receptors, such as integrins [19, 20]. Although its role in sarcoidosis pathogenesis remains largely unknown, emerging evidence suggests its involvement in sarcoid granuloma formation. In pulmonary arterial hypertension, macrophage‐derived LGMN activates MMP‐2 via proteolytic removal of an N‐terminal propeptide region, whereas elevated MMP‐2 levels have been confirmed in sarcoidosis [36, 37]. Additionally, LGMN is upregulated in various tumors and plays a crucial role in the polarization of TAMs [22]. Furthermore, LGMN drives Th1 induction in human CD4^+^ T cells [38] and may impair regulatory T cell differentiation and function by facilitating TRAF6 degradation [39]. Given the critical roles of macrophage polarization, Th1 activation, and regulatory T cell dysfunction in sarcoidosis [6], we hypothesized that LGMN contributes to the sarcoidosis immunopathogenesis. Our in vivo and in vitro experiments confirmed the upregulation of LGMN in sarcoid granulomas and its predominant expression in macrophages. Moreover, *Lgmn* deletion promoted granuloma formation, highlighting its potential role in modulating granulomatous inflammation.

Macrophage polarization remains a controversial topic in sarcoidosis research. Early studies have reported elevated levels of proinflammatory cytokines, including tumor necrosis factor alpha (TNF‐α), IFN‐γ, IL‐6, IL‐1β, and CXCL10, in patients with sarcoidosis [40, 41]. Sarcoid granulomas activate the pentose–phosphate pathway, which supports M1‐like macrophage survival by rapidly replenishing NADPH and maintaining redox homeostasis [9, 42]. Notably, pentose–phosphate pathway inhibition effectively suppressed granuloma formation in both in vitro and in vivo models of granuloma [9]. These findings emphasize the pivotal role of M1 polarization in driving sarcoid granulomatous inflammation. However, growing evidence suggests that M2 polarization may be essential, especially in the advanced stages of sarcoidosis [8, 15, 43]. For instance, IL‐13‐driven M2 polarization via STAT6 activation promotes granuloma formation in purified protein derivative‐stimulated sarcoid peripheral blood mononuclear cells [15]. In the present study, elevated expression of both M1 and M2 macrophage polarization markers was observed in the lymph nodes of patients with sarcoidosis, as well as in the lungs of mice challenged with *P. acnes* and TDM. Deletion of *Lgmn* exacerbated *P. acnes*‐induced granulomatous inflammation concomitant with enhanced M1 polarization, highlighting the contribution of M1‑driven immune responses to granulomatous inflammation. In contrast, the levels of M2 polarization markers remained largely unchanged. These findings suggest that the observed increase in M2 polarization may reflect an adaptive response aimed at limiting tissue injury within the inflammatory milieu of sarcoidosis, rather than serving as a principal driver of granuloma formation. This interpretation is further supported by our independent validation in the TDM‐induced mouse model, where LGMN overexpression attenuated granulomatous inflammation and reduced M1‐associated markers. Together, these results suggest that M1 polarization plays a central role in driving sarcoid‐like granulomatous inflammation, while M2 activation more likely represents a compensatory response.

Previous studies have demonstrated that LGMN is highly expressed in macrophages, particularly M2‐like macrophages, and contributes to M2 polarization under diverse pathological conditions [22, 26]. Consistent with these findings, we observed upregulated LGMN expression in M2‐like BMDMs stimulated with IL‐4 and IL‐13. However, *Lgmn* deficiency did not impair M2 polarization in the *P. acnes*‐induced mouse model, indicating that LGMN is dispensable for maintaining M2 polarization in the context of sarcoid granulomatous inflammation. Notably, exogenous LGMN has been reported to exert anti‐inflammatory effects. For instance, in the tumor microenvironment, exogenous LGMN reprogrammed M1‐like macrophages toward an M2 phenotype via PI3K/Akt/mTORC2 signaling pathway activation [25]. In the current study, although LGMN was preferentially expressed in M2‐like macrophages, its deficiency predominantly promoted M1 polarization, which was accompanied by enhanced mTORC1/STAT1 activation and associated with exacerbated granulomatous inflammation. Taken together, within the inflammatory setting of sarcoidosis, LGMN may not act as a critical driver of M2 polarization but rather as an anti‐inflammatory mediator secreted by M2‐like macrophages. As part of the adaptive anti‐inflammatory response, LGMN mainly functions to restrain excessive M1 polarization, thereby maintaining immune homeostasis and contributing to the resolution of granulomatous inflammation.

LGMN possesses a distinctive integrin‐binding RGD motif, enabling its interaction with integrin αvβ3 on the cell surface [44]. Previous studies have demonstrated that LGMN–integrin αvβ3 interaction plays a multifaceted role, including promoting cancer cell migration and invasion, inhibiting vascular smooth muscle cell differentiation, and suppressing STAT1‐dependent IL‐1β secretion [19, 20, 23]. Consistently, our data confirmed that LGMN interacts with integrin αvβ3 within sarcoid‐like granulomas. Treatment with the integrin αvβ3 antagonist Cyclo(‐RGDfK) led to significant upregulation of iNOS expression in BMDMs, suggesting that LGMN–integrin αvβ3 interaction mediates the suppression of M1 polarization. However, the mechanism by which LGMN–integrin αvβ3 interaction suppresses mTORC1/STAT1 activation remains unclear, requiring further investigation. Additionally, early studies have shown that integrin αvβ3 binding to other ligands, such as Tenascin‐C and ANGPTL13, enhances inflammatory responses by activating NF‐κB signaling in macrophages [45, 46]. It is plausible that the ligands engaging with integrin αvβ3, rather than the receptor itself, exert a more decisive influence on macrophage polarization.

STAT1 is a well‐established key regulator of M1 polarization programming [28]. Its full activation requires phosphorylation at both Tyr701 and Ser727, which can be induced by FcγRs, TNF‐α, and IFN‐γ signaling [47, 48, 49]. IFN‐γ‐induced STAT1 phosphorylation at Tyr701 is JAK1/JAK2‐dependent and is essential for STAT1 dimerization and nuclear translocation. Additionally, STAT1 phosphorylation at Ser727, which regulates its transcriptional activity, occurs independently of JAK1/JAK2 and can be activated by p38 MAPK and PKC‐δ [48, 49, 50]. JAK/STAT1 pathway activation has been reported in both tissue and blood samples from patients with sarcoidosis. An increasing number of studies have focused on JAK inhibition in sarcoidosis, demonstrating the efficacy of JAK inhibitors such as tofacitinib (JAK1/3> JAK2 inhibitor) and ruxolitinib (JAK1/2 inhibitor) [51, 52, 53, 54, 55, 56]. LGMN deficiency in TAMs has been reported to sustain STAT1 activation, enhance IL‐1β production, and promote tumor cell senescence [23]. Consistent with this, our data demonstrated that *Lgmn* deficiency increased STAT1 phosphorylation at both Tyr701 and Ser727, further driving M1 polarization. Notably, the upregulation of JAK1 phosphorylation was not significant in the current mouse model, suggesting that STAT1 activation may be mediated by alternative signaling pathways, such as p38 MAPK, which has been implicated in sarcoidosis [57, 58]. Given the persistent STAT1 activation in sarcoid granulomas and its pivotal role in M1 polarization, therapeutic strategies aimed at modulating STAT1 at the transcriptional level, phosphorylation sites, or dimerization interfaces may provide a more effective approach for sarcoidosis treatment, especially in patients insensitive to JAK inhibitors. In the present study, treatment with *Lgmn* pDNA‐loaded LNPs significantly upregulated LGMN protein expression and alleviated both *P. acnes*‐ and TDM‐induced pulmonary granulomatous inflammation. The associated reduction in STAT1 phosphorylation highlights the therapeutic potential of LGMN modulation for regulating STAT1‐driven inflammation in sarcoidosis.

mTORC1 functions as a central integrator of intra‐ and extracellular signals to regulate cellular metabolism and has been implicated in sarcoid granuloma formation [8]. Integrin αvβ3 has also been reported to modulate mTORC1 activation across multiple cell types [59, 60]. In the present study, we found that LGMN interacts with integrin αvβ3 and that *Lgmn* deficiency promotes mTORC1 activation. These findings raise the possibility that LGMN may restrain mTORC1 activation through its interaction with integrin αvβ3, which warrants further investigation. We next examined the relationship between mTORC1 and STAT1 signaling. Prior studies have described crosstalk between these pathways in immune cells, including the MST1–mTORC1–STAT1 axis in B cells and IL‑2–mTORC1–STAT1 signaling in T cells [61, 62, 63]. In our study, pharmacologic inhibition of mTORC1 with Rapa reduced STAT1 phosphorylation in BMDMs, whereas the STAT1 inhibitor F‐ara‐A had minimal effect on p‑S6 expression. These findings indicate that mTORC1 likely functions upstream of STAT1 in M1‑like macrophages. Given the established role of mTORC1 in glucose metabolism, we also examined the impact of *Lgmn* deficiency on glycolysis, which is known to be upregulated in sarcoid granulomas to support macrophage proliferation [8, 14]. Consistent with this metabolic shift, *Lgmn*‐KO BMDMs undergoing M1 polarization exhibited reduced OCR. However, in vivo, *Lgmn* deficiency resulted in only modest alterations in the expression of glycolysis‑related genes. This divergence between in vitro and in vivo findings may reflect the complex cellular composition of granulomatous lesions or the presence of compensatory metabolic mechanisms at the tissue level.

Prednisone remains the cornerstone therapy for both pulmonary and extrapulmonary sarcoidosis, although its use is often limited by its broad spectrum of side effects [4]. Second‐line agents, including Methotrexate and Azathioprine, have been reported to induce hematologic and hepatic toxicities [64]. Compared with the abovementioned systemic therapies, LNPs administered via inhalation have shown significant advantages in minimizing toxicity in non‐pulmonary organs [65]. In the current study, *Lgmn* pDNA‐loaded LNPs exhibited significant therapeutic efficacy in two distinct sarcoid‐like granulomatous mouse models. Additionally, IVIS imaging revealed a strong fluorescent signal restricted to the lungs after intratracheal injection that persisted for over 6 days, indicating its excellent lung‐targeting specificity with prolonged retention. These results suggest the therapeutic potential of *Lgmn* pDNA‐loaded LNPs for the treatment of pulmonary sarcoidosis in clinical practice.

The present study has some limitations. First, we used whole‐body *Lgmn*‐KO mice to examine the protective role of LGMN in sarcoid granulomas. Despite the predominant expression of LGMN in macrophages, it can also be detected in other immune cells, such as dendritic cells and T cells [38, 39]. Consequently, utilizing conditional KO mice may provide a more precise approach to investigate the specific role of macrophage‐derived LGMN. Second, our findings demonstrated that LGMN suppresses mTORC1/STAT1‐mediated M1 polarization through its interaction with integrin αvβ3. However, the precise mechanisms underlying these processes were not elucidated in the present study and require further investigation. Third, although we additionally measured LGMN levels in BALF samples from patients with sarcoidosis, the lack of healthy controls and the absence of serum measurements still limit our ability to assess the correlation between LGMN levels and disease severity.

## 4 Conclusions

This study demonstrated that LGMN is highly expressed in macrophages within sarcoid granulomas, and *Lgmn* deficiency exacerbates *P. acnes*‐induced lung granuloma formation by promoting M1 polarization. We found that LGMN interacts with integrin αvβ3 and further inhibits mTORC1/ STAT1 signaling, thereby suppressing M1 polarization in granuloma formation. Furthermore, intratracheal delivery of *Lgmn* pDNA‐loaded LNPs significantly attenuated sarcoid‐like granuloma inflammation in mice. Collectively, these findings suggest that LGMN supplementation is a promising therapeutic approach for mitigating granulomatous inflammation in sarcoidosis.

## 5 Experimental Section

### 5.1 Human Samples

All human samples were obtained at the China‐Japan Friendship Hospital. BALF samples were obtained from sarcoidosis patients (n = 50). For histological analysis, formalin‐fixed paraffin‐embedded (FFPE) tissue sections were used, including lung tissues from sarcoidosis patients (n = 5) and control subjects (n = 4), and lymph node tissues from sarcoidosis patients (n = 5). Fresh frozen lymph node tissues were also collected from sarcoidosis patients (n = 3) and lung transplant donors (n = 3). Diagnoses were established using a multimodal approach, incorporating clinical, radiological, and BAL analyses, supported by histological evidence of non‐caseating granulomas, according to the American Thoracic Society/European Respiratory Society/World Association for Sarcoidosis and Other Granulomatous Disorders statement [66]. The clinical information of the patients with sarcoidosis is summarized in Table S1. The study protocols were approved by the Medical Ethics Committee of China‐Japan Friendship Hospital (2017–25), and written informed consent was obtained from all participants.

BALF samples from sarcoidosis patients were centrifuged at 2000 rpm for 10 min, and the supernatants were collected and stored at −80°C until analysis. LGMN levels in BALF were measured using a human LGMN ELISA kit (Abcam, Cambridge, UK) according to the manufacturer's instructions. Absorbance was recorded at 450 nm using a TECAN SPARK multimode microplate reader (Tecan Group Ltd., Männedorf, Switzerland), and LGMN concentrations were calculated from a standard curve.

### 5.2 Animals Experiments

WT C57BL/6 female mice (8–10 weeks old) were obtained from Vital River Laboratories (Beijing, China). *Lgmn*‐knockout mice (*Lgmn*‐KO; C57BL/6 background; strain No. T011664) were purchased from GemPharmatech Co. Ltd. (Nanjing, China). *Lgmn* depletion was confirmed by genotyping of tail blood DNA. All animals were maintained under specific pathogen‐free conditions at the China‐Japan Friendship Hospital. Animal experiments were conducted according to protocols approved by the Institutional Animal Care and Use Committee of the China‐Japan Friendship Hospital (ZRDWLL240118).

Pulmonary granulomas were induced in female WT and *Lgmn*‐KO mice using *P. acnes* (American Type Culture Collection, Manassas, VA, USA). Mice were first sensitized by intraperitoneal injection of 25 mg/kg *P. acnes* suspension. On day 14, mice received an intratracheal challenge with either 50 or 75 mg/kg *P. acnes*. Control mice received an equal volume of sterile PBS. Two therapeutic experiments were performed. For early intervention, mice challenged with 50 mg/kg *P. acnes* were treated with *Lgmn* pDNA–loaded LNPs (1 mg/kg) on day 14 after sensitization. For treatment of established granulomas, mice received 75 mg/kg *P. acnes* on day 14 followed by LNP administration (1 mg/kg) on day 17. Finally, the mice were sacrificed on day 21 after the initial intraperitoneal injection.

TDM (MedChemExpress, Shanghai, China) was also used to induce pulmonary granuloma formation in female WT mice. TDM was prepared in a water‐in‐oil emulsion using incomplete Freund's adjuvant (IFA; Sigma‐Aldrich, Merck KGaA, Darmstadt, Germany) as previously described [67]. 1 µg TDM was dissolved in 5 µL of the emulsion (IFA/PBS and 0.2% Tween80), and administered intravenously via the tail vein at a dose of 1.25 mg/kg. Control mice received an equal volume of vehicle. To evaluate the therapeutic effects of LGMN, WT mice received intratracheal administration of *Lgmn* pDNA‐loaded LNPs (1 mg/kg) on day 0 after TDM injection. Lungs were harvested on day 7 to assess granuloma formation.

### 5.3 Histological Analyses and IHC

The left lungs of the mice were fixed in 4% paraformaldehyde for 24 h and subsequently embedded in paraffin. The paraffin‐embedded lung tissues were sectioned at a thickness of 3 µm and subjected to H&E staining. IHC was performed on 3 µm paraffin‐embedded tissue sections using the following primary antibodies: anti‐CD68 (Proteintech, Wuhan, China), anti‐CD4 (Abcam, Cambridge, UK), anti‐Mac2 (Cedarlane, Burlington, Canada), anti‐iNOS (Abcam, Cambridge, UK), and anti‐Arg1(Proteintech, Wuhan, China). Tissue sections were incubated with primary antibodies overnight at 4°C, followed by incubation with secondary antibodies for 1 h at room temperature. The sections were then stained with DAB reagent (Zhong Shan Golden Bridge Biological Technology Co., Ltd., Beijing, China) according to the manufacturer's instructions, and cell nuclei were counterstained with hematoxylin. Stained cells were examined under an optical light microscope.

For histological evaluation, serial lung sections were stained with H&E and subjected to IHC staining for CD4 and Mac‐2, with CD68 included when available. Whole‐slide images were acquired using a Pannoramic MIDI Digital Scanner (3DHISTECH, Budapest, Hungary). For granuloma area quantification in the *P. acnes*‐induced mouse model, well‐defined granulomas were manually delineated using the annotation tools in CaseViewer (3DHISTECH, Budapest, Hungary). At least five granulomas per section were measured, and areas were automatically calculated by the software. For the TDM‐induced model, granuloma burden per lung section was assessed as previously described [67].

### 5.4 Immunofluorescence

Following routine dewaxing, hydration, antigen retrieval, and blocking of non‐specific binding sites, lymph node and lung tissue sections were incubated overnight at 4°C with primary antibodies, including anti‐LGMN (Cell Signaling Technology, Danvers, MA, USA), anti‐Mac2 (Cedarlane, Burlington, Canada), anti‐Arg1 (Proteintech, Wuhan, China), anti‐pS6 (Cell Signaling Technology, Danvers, MA, USA), and anti‐pSTAT1 (Ser727) (Cell Signaling Technology, Danvers, MA, USA). Tissue sections were subsequently stained with Alexa Fluor 488‐labeled, Cy3‐conjugated, or Cy7‐conjugated donkey anti‐rabbit/rat antibodies (Jackson ImmunoResearch Laboratories, West Grove, PA, USA). Nuclei were counterstained with DAPI. Images were captured and analyzed by confocal fluorescence microscopy (Leica Microsystems, Wetzlar, Germany) or Pannoramic MIDI Digital Scanner.

### 5.5 Cell Culture and Treatment

To obtain mouse BMDMs, the tibia and femur were carefully isolated from C57BL/6J mice under sterile conditions. Both ends of the bones were excised, and the bone marrow cavity was thoroughly flushed with Dulbecco's Modified Eagle Medium (DMEM; KeyGEN Biotech, Nanjing, China) until complete transparency was achieved. The harvested cells were filtered through a 70‐µm cell strainer to remove debris. To eliminate erythrocytes, the cell suspension was treated with red blood cell lysis buffer. Following centrifugation, the pelleted cells were resuspended and cultured in DMEM supplemented with 15% fetal bovine serum (FBS) and 50 ng/mL recombinant mouse macrophage colony‐stimulating factor (M‐CSF) (Novoprotein Scientific Inc., Shanghai, China). On days 3 and 5, half of the culture medium was replaced with fresh DMEM containing 50 ng/mL M‐CSF to promote macrophage differentiation. On day 7, the differentiated BMDMs were subjected to different stimulation conditions. For M1 polarization, BMDMs were treated with 50 ng/mL LPS (Sigma‐Aldrich, Merck KGaA, Darmstadt, Germany) and 20 ng/mL recombinant mouse IFN‐γ (PeproTech, Cranbury, NJ, USA) and harvested at the indicated time points (0, 3, 6, and 12 h) for time‐course experiments, or at 12 h for all other experiments. For M2 polarization, BMDMs were treated with 20 ng/mL recombinant mouse IL‐4 (PeproTech, Cranbury, NJ, USA) and 20 ng/mL recombinant mouse IL‐13 (PeproTech, Cranbury, NJ, USA) for 24 or 48 h. For the indicated experiments, 10 µM Cyclo(‐RGDfK) (MedChemExpress, Shanghai, China), 1 µM Rapa (MedChemExpress, Shanghai, China), or 50 µM F‐ara‐A (MedChemExpress, Shanghai, China) was added during M1 polarization.

RAW264.7 cells (American Type Culture Collection, Manassas, VA, USA) were cultured in DMEM supplemented with 10% FBS. HEK‐293T cells (Procell Life Science & Technology Co., Ltd., Wuhan, China) were maintained in DMEM containing 10% FBS and a 1:100 dilution of an antibiotic–antimycotic solution. All cell cultures were maintained at 37°C in a humidified atmosphere containing 5% CO_2_. To generate M1‐like macrophages, RAW264.7 cells were stimulated by 50 ng/mL LPS and 20 ng/mL IFN‐γ. After 12 h of incubation, the cells were harvested for subsequent analysis.

### 5.6 Plasmid Transfection

*Lgmn* and vector plasmids were purchased from Shanghai GeneChem Co., Ltd. (Shanghai, China). A total of 7.5 µg of purified DNA was transfected into RAW264.7 and HEK‐293T cells using Lipofectamine 3000 transfection reagent (Thermo Fisher Scientific, Waltham, MA, USA) according to the manufacturer's protocols. Transfection efficiency was assessed 48 h post‐transfection by Western blot analysis. After 36 h of transfection, RAW264.7 cells were stimulated with 50 ng/mL LPS and 20 ng/mL IFN‐γ for 12 h. The cells were collected for subsequent experiments.

### 5.7 OCR Detection

After treatment according to the experimental grouping, BMDMs were cultured for 12 h, and the medium was then discarded. OCR was measured using a commercial assay kit (E‐BC‐F070, Elabscience Biotechnology, Wuhan, China) following the manufacturer's instructions. The microplate was placed in a TECAN SPARK multimode microplate reader maintained at 37°C. Fluorescence was recorded at excitation/emission wavelengths of 405/650 nm every 2 min for 120 min. Fluorescence values were plotted against time, and OCR was calculated as the slope of the resulting curves.

### 5.8 Western Blot

Total protein was extracted from lung tissues, lymph node tissues, and cultured cells using radioimmunoprecipitation assay lysis buffer (Solarbio, Beijing, China) containing protease inhibitors (MedChemExpress, Shanghai, China), phosphatase inhibitors (MedChemExpress, Shanghai, China), and phenylmethylsulfonyl fluoride (Solarbio, Beijing, China). The lysates were collected and centrifuged at 13 000 rpm for 20 min, and the protein concentration was determined using a bicinchoninic acid (BCA) protein assay kit (Beyotime, Shanghai, China) according to the manufacturer's protocol. After mixing with loading buffer and heating at 100°C for 10 min, 30–50 µg of protein was separated by 10% sodium dodecyl sulfate (SDS)–polyacrylamide gel electrophoresis and subsequently transferred to polyvinylidene difluoride (PVDF) membranes (Millipore, Billerica, MA, USA). The membranes were blocked with 5% skimmed milk for 2 h and incubated overnight at 4°C with the following primary antibodies: anti‐LGMN (Abcam, Cambridge, UK), anti‐iNOS (Abcam, Cambridge, UK), anti‐Arg1 (Proteintech, Wuhan, China), anti‐CD206 (Proteintech, Wuhan, China), anti‐STAT1 (Abcam, Cambridge, UK), anti‐pSTAT1(Tyr701) (Cell Signaling Technology, Danvers, MA, USA), anti‐pSTAT1(Ser727) (Cell Signaling Technology, Danvers, MA, USA), anti‐JAK1 (Proteintech, Wuhan, China), anti‐pJAK1(Tyr1034/1035) (Cell Signaling Technology, Danvers, MA, USA), anti‐ITGAV (Abcam, Cambridge, UK), anti‐ITGB3 (Abcam, Cambridge, UK), anti‐pS6 (Cell Signaling Technology, Danvers, MA, USA), anti‐S6 (Cell Signaling Technology, Danvers, MA, USA), anti‐HK2 (Cell Signaling Technology, Danvers, MA, USA), anti‐LDHA (Proteintech, Wuhan, China), and anti‐β‐actin (Proteintech, Wuhan, China). Following three washes in Tris‐buffered saline containing 0.1% Tween 20 (TBST), the PVDF membranes were incubated with the corresponding secondary antibodies for 1 h and washed again in TBST three times. Protein detection was performed using a chemiluminescent substrate system (Bio‐Rad Laboratories, Hercules, CA, USA), and gray values were analyzed using ImageJ software (Version 1.53k, USA).

### 5.9 qRT‐PCR

Total RNA was isolated from the mouse lung tissues using RNAiso PLUS reagent (Takara Bio Engineering (Dalian) Co., Ltd., Dalian, China) and reverse‐transcribed into cDNA using Hifair III 1st Strand cDNA Synthesis SuperMix (Yeasen Biotechnology (Shanghai) Co., Ltd., Shanghai, China). cDNA was used for RT‐PCR amplification using Hieff qPCR SYBR Green Master Mix (Yeasen Biotechnology (Shanghai) Co., Ltd., Shanghai, China). The relative quantification of mRNA expression was calculated using the 2^−∆∆Ct^ method after normalization to *Actb*. The primers used to amplify each target gene are listed in Table S2.

### 5.10 Co‐Immunoprecipitation

Total protein was extracted from the lung tissues using lysis buffer, and the protein concentration was determined using a BCA protein assay kit, following the manufacturer's protocol. One milligram of total protein was used for subsequent immunoprecipitation assays. Each sample was incubated overnight at 4°C with 1 µg of primary antibody targeting LGMN (Santa Cruz Biotechnology, Inc., Dallas, TX, USA). Mouse immunoglobulin G (Beyotime, Shanghai, China) was used as a negative control. Subsequently, lysates were incubated with 30 µL of Protein A/G Beads (MedChemExpress, Shanghai, China) at 4°C for 2 h. The immunocomplexes were washed five times with lysis buffer, resuspended in 2 × SDS loading buffer, and heated at 95°C for Western blot analysis.

### 5.11 Synthesis and Characterization of LNPs

The pDNA‐loaded LNPs were generated as previously reported [68, 69]. A lipid solution in which SM102, cholesterol, DSPC, and PEG‐DMG‐2K were dissolved in ethanol at a molar ratio of 57.2:24.0:12.7:6.1 was prepared. pDNA was dissolved in PBS, and the lipid components were mixed with the pDNA solution at a weight ratio of 1:3 using the NanoGenerator Flex‐M system (PreciGenome LLC, San Jose, CA, USA). The prepared LNPs were subsequently ultracentrifuged to remove the residual ethanol and free pDNA. Finally, pDNA‐loaded LNPs were diluted in PBS. The hydrodynamic diameter, PDI, and zeta potential of the LNPs were measured by dynamic light scattering using a Zetasizer Nano‐ZS (Malvern Panalytical Ltd., Malvern, UK). The amount of encapsulated pDNA carried by the LNPs was evaluated using a Quanti‐iT PicoGreen dsDNA assay kit (ThermoFisher Scientific, Waltham, MA, USA). After staining with 2% phosphotungstic acid, the structure of the LNPs was characterized by TEM (JEOL Ltd., Tokyo, Japan).

### 5.12 In Vivo Biodistribution of LNPs

DiR‐labeled LNPs were prepared as previously reported [70]. The mice were treated with DiR‐loaded LNPs via intratracheal injection 14 d after *P. acnes* sensitization. The mice were anesthetized with isoflurane (Sigma‐Aldrich, Merck KGaA, Darmstadt, Germany) and imaged at specific time points (0, 1, 3, and 6 days) using an IVIS (AniView Phoenix, Biolight Biotechnology, Guangzhou, China) with the following parameters: excitation, 745 nm; emission, 820 nm. On day 3 after intratracheal delivery, the mice were euthanized, and the organs were removed for ex vivo fluorescence imaging.

### 5.13 In Vivo Toxicity Studies of LNPs

Mice without *P. acnes* challenge were randomly assigned into four groups and received intratracheal delivery of *Lgmn* pDNA‐loaded LNPs (1 mg/kg). Major organs were collected for histopathological analysis on days 0, 1, 3, and 6 following LNP administration. For the early‑intervention experiment, mice challenged with 50 mg/kg *P. acnes* were treated with *Lgmn* pDNA–loaded LNPs (1 mg/kg) on day 14 after sensitization. On day 21 after *P. acnes* sensitization, blood and urine samples were collected for biochemical analyses of liver and kidney function, as well as urinalysis.

### 5.14 Analysis of Public Bulk RNA‐seq Data

The raw transcriptome data used in this study were retrieved from the Gene Expression Omnibus (GEO; https://www.ncbi.nlm.nih.gov/geo/) database. The GSE73394 dataset contains BAL cell data from 26 patients with sarcoidosis and 20 healthy donors, whereas the GSE75023 dataset includes BAL cell data from 15 patients with sarcoidosis and 12 healthy controls. The GEOquery package (version 2.66.0) was used to download the series matrix files from the GEO database in R (version 4.2.2). DEGs between patients with sarcoidosis and healthy donors were identified using the limma package (version 3.54.2). Significant DEGs were identified according to the thresholds of adjusted *p* < 0.05 and fold change > 2 or < 0.5. Common DEGs across the datasets were visualized using the R packages ggplot2 (version 3.5.1) and tidyverse (version 2.0.0).

To evaluate the diagnostic value of LGMN in pulmonary sarcoidosis, the GSE109516 dataset, containing BAL cell transcriptome data from 199 patients with sarcoidosis, was obtained from the GEO database and used as the discovery cohort. Fragments per kilobase of transcript per million mapped reads (FPKM) values were converted to TPM. Based on the Scadding stage, patients with stage 0–I were classified as the non‐parenchymal involvement group, and those with stage II–IV as the parenchymal involvement group. LGMN expression was compared between the two groups using two approaches: the Wilcoxon rank‐sum test was applied to TPM values, and the unpaired Student's *t*‐test was applied to log2‐transformed (TPM + 1) values. The optimal cutoff value for identifying parenchymal involvement was determined using the Youden index derived from receiver operating characteristic (ROC) curve analysis, followed by internal cross‑validation to refine the threshold. To assess the robustness and generalizability of this threshold, the external dataset GSE73394 was used for validation. In this dataset, diagnostic performance was assessed using two strategies: direct application of the previously established cutoff and independent threshold determination by percentile matching. Sensitivity, specificity, and AUC were calculated using R (version 4.2.2).

### 5.15 Analysis of Public scRNA‐seq Data

scRNA‐seq data were retrieved from the GEO database. The GSE227136 dataset included human lung tissue samples from 116 individuals, comprising 67 patients with pulmonary fibrosis and 49 healthy donors. The data of four patients with sarcoidosis and five age‐ and sex‐matched controls were selected for subsequent analysis. The GSE250508 dataset contains skin‐cell data from *Tsc2* ^fl/fl^ CD11c‐Cre ^+^ sarcoidosis mice and *Tsc2* ^fl/fl^ controls (n = 3 per group). Cluster information was obtained directly from the database. Data analysis and visualization were performed using the R package Seurat (version 4.5.0).

### 5.16 Statistical Analysis

For continuous variables, data are presented as mean ± standard error of the mean (SEM) and were analyzed and visualized using GraphPad Prism software (version 8.0, USA). The sample size (n) represents the number of human biopsies per group, the number of mice per group, or the number of replicates for ex vivo cell experiments. Statistical comparisons between two experimental groups were performed using an unpaired *t*‐test, whereas one‐way analysis of variance (ANOVA) was used to assess statistical differences among three or more groups. For categorical variables, Fisher's exact test (Freeman–Halton extension for tables larger than 2 × 2) was applied. Statistical significance was set at *p* < 0.05.

## Author Contributions

Mengyuan Liu: study design, experiments, data acquisition and analysis, and manuscript drafting and revision. Yueyin Han: experiments, data acquisition, and manuscript revision. Bingbing Xie: conception, clinical data collection, and result interpretation. Lili Zhu: clinical data collection and result interpretation. Wenxiu Xu: experiments and data analysis. Yinzhen Han: experiments and data analysis. Yue Liao: experiments and data analysis. Shuwei Gao: experiments and data analysis. Dingyuan Jiang: supervision, data collection, and result interpretation. Jing Geng: supervision, data collection, and result interpretation. Zhen Li: study design, supervision, experiments, data acquisition and analysis, and critical manuscript revision. Yinan Hu: study design, supervision, data acquisition and analysis, and critical manuscript revision. Huaping Dai: study conception, supervision, result interpretation, critical manuscript revision, and the final approval of the version to be published. All authors contributed to the article and approved the submitted version.

## Conflicts of Interest

The authors declare no conflict of interest.

## Funding

This study was supported by National Natural Science Foundation of China (grant number 82500089, 82370072, and 82170080), Capital's Funds for Health Improvement and Research (grant number 2024‐1‐4062), Beijing Nova Program (grant number 20230484281), State Key Laboratory Special Fund (grant number 2060204), and National High Level Hospital Clinical Research Funding (grant number BJ‐2024‐251).

## Supporting information




**Supporting File**: advs75043‐sup‐0001‐SuppMat.pdf.

## Data Availability

The transcriptomic datasets that support the findings of this study are publicly available in the Gene Expression Omnibus (GEO) database. The accession numbers are GSE73394, GSE75023, GSE109516, GSE227136, and GSE250508. These datasets can be accessed via https://www.ncbi.nlm.nih.gov/geo/.

## References

[1] M. Drent , E. D. Crouser , and J. Grunewald , “Challenges of Sarcoidosis and Its Management,” New England Journal of Medicine 385, no. 11 (2021): 1018–1032, 10.1056/NEJMra2101555. 34496176

[2] J. A. Belperio , F. Shaikh , F. G. Abtin , et al., “Diagnosis and Treatment of Pulmonary Sarcoidosis,” Jama 327, no. 9 (2022): 856, 10.1001/jama.2022.1570. 35230389

[3] J. S. Kim and R. Gupta , “Lung Transplantation in Pulmonary Sarcoidosis,” Journal of Autoimmunity 149 (2024): 103135, 10.1016/j.jaut.2023.103135. 37923622

[4] F. F. Rahaghi , R. P. Baughman , L. A. Saketkoo , et al., “Delphi Consensus Recommendations for a Treatment Algorithm in Pulmonary Sarcoidosis,” European Respiratory Review 29, no. 155 (2020): 190146, 10.1183/16000617.0146-2019. 32198218 PMC9488897

[5] M. A. Judson , “Causes of Poor Medication Adherence in Sarcoidosis,” Chest 158, no. 1 (2020): 17–18, 10.1016/j.chest.2020.03.001. 32654702

[6] J. Miedema , F. Cinetto , A. Smed‐Sörensen , and P. Spagnolo , “The Immunopathogenesis of Sarcoidosis,” Journal of Autoimmunity 149 (2024): 103247, 10.1016/j.jaut.2024.103247. 38734536

[7] T. Goldmann , G. Zissel , R. S. Gupta , M. Schlaak , E. Vollmer , and J. Müller‐Quernheim , “Formation of Granulomas in the Lungs of Severe Combined Immunodeficient Mice After Infection With bacillus Calmette‐Guerin,” The American Journal of Pathology 158, no. 5 (2001): 1890–1891, 10.1016/s0002-9440(10)64147-8. 11337389 PMC1891941

[8] M. Linke , H. T. T. Pham , K. Katholnig , et al., “Chronic Signaling via the Metabolic Checkpoint Kinase mTORC1 Induces Macrophage Granuloma Formation and Marks Sarcoidosis Progression,” Nature Immunology 18, no. 3 (2017): 293–302, 10.1038/ni.3655. 28092373 PMC5321578

[9] S. Nakamizo , Y. Sugiura , Y. Ishida , et al., “Activation of the Pentose Phosphate Pathway in Macrophages Is Crucial for Granuloma Formation in Sarcoidosis,” Journal of Clinical Investigation 133, no. 23 (2023): 171088, 10.1172/jci171088. PMC10688990 38038136

[10] Y. Sun , X. Gu , E. Zhang , et al., “Estradiol Promotes Pentose Phosphate Pathway Addiction and Cell Survival via Reactivation of Akt in mTORC1 Hyperactive Cells,” Cell Death & Disease 5, no. 5 (2014): 1231, 10.1038/cddis.2014.204. PMC4047866 24832603

[11] M. Silic‐Benussi , E. Sharova , A. Corradin , et al., “Repurposing Verapamil to Enhance Killing of T‐ALL Cells by the mTOR Inhibitor Everolimus,” Antioxidants (Basel) 12, no. 3 (2023): 625, 10.3390/antiox12030625. 36978873 PMC10045900

[12] H. R. Griffiths , D. Gao , and C. Pararasa , “Redox Regulation in Metabolic Programming and Inflammation,” Redox Biology 12 (2017): 50–57, 10.1016/j.redox.2017.01.023. 28212523 PMC5312548

[13] P. Wojtan , M. Mierzejewski , I. Osińska , and J. Domagała‐Kulawik , “Macrophage Polarization in Interstitial Lung Diseases,” Central European Journal of Immunology 41, no. 2 (2016): 159–164, 10.5114/ceji.2016.60990. 27536201 PMC4967650

[14] R. A. Gonçales , H. N. Bastos , C. Duarte‐Oliveira , et al., “Pentraxin 3 Inhibits Complement‐Driven Macrophage Activation to Restrain Granuloma Formation in Sarcoidosis,” American Journal of Respiratory and Critical Care Medicine 206, no. 9 (2022): 1140, 10.1164/rccm.202112-2771OC. 35767663 PMC9704840

[15] L. W. Locke , E. D. Crouser , P. White , et al., “IL‐13–Regulated Macrophage Polarization During Granuloma Formation in an In Vitro Human Sarcoidosis Model,” American Journal of Respiratory Cell and Molecular Biology 60, no. 1 (2019): 84–95, 10.1165/rcmb.2018-0053OC. 30134122 PMC6348723

[16] M. H. Haugen , H. T. Johansen , S. J. Pettersen , et al., “Nuclear Legumain Activity in Colorectal Cancer,” PLoS ONE 8, no. 1 (2013): 52980, 10.1371/journal.pone.0052980. PMC3542341 23326369

[17] R. Maehr , H. C. Hang , J. D. Mintern , et al., “Asparagine Endopeptidase Is Not Essential for Class II MHC Antigen Presentation but Is Required for Processing of Cathepsin L in Mice,” The Journal of Immunology 174, no. 11 (2005): 7066–7074, 10.4049/jimmunol.174.11.7066. 15905550

[18] Z. Zhang , Y. Tian , and K. Ye , “δ‐Secretase in Neurodegenerative Diseases: Mechanisms, Regulators and Therapeutic Opportunities,” Translational Neurodegeneration 9 (2020): 1, 10.1186/s40035-019-0179-3. 31911834 PMC6943888

[19] C. Liu , J. Wang , Y. Zheng , et al., “Autocrine Pro‐Legumain Promotes Breast Cancer Metastasis via Binding to Integrin αvβ3,” Oncogene 41, no. 34 (2022): 4091–4103, 10.1038/s41388-022-02409-4. 35854065

[20] L. Pan , P. Bai , X. Weng , et al., “Legumain Is an Endogenous Modulator of Integrin αvβ3 Triggering Vascular Degeneration, Dissection, and Rupture,” Circulation 145, no. 9 (2022): 659–674, 10.1161/circulationaha.121.056640. 35100526

[21] X. Meng , B. Li , M. Wang , W. Zheng , and K. Ye , “Development of Asparagine Endopeptidase Inhibitors for Treating Neurodegenerative Diseases,” Trends in Molecular Medicine 31, no. 4 (2025): 359–372, 10.1016/j.molmed.2025.01.009. 40000317

[22] L. Pang , S. Guo , F. Khan , et al., “Hypoxia‐Driven Protease Legumain Promotes Immunosuppression in Glioblastoma,” Cell Reports Medicine 4, no. 11 (2023): 101238, 10.1016/j.xcrm.2023.101238. 37858339 PMC10694605

[23] L. Shen , L. Kang , D. Wang , et al., “Legumain‐deficient Macrophages Promote Senescence of Tumor Cells by Sustaining JAK1/STAT1 Activation,” Cancer Letters 472 (2020): 40–49, 10.1016/j.canlet.2019.12.013. 31857155

[24] D. Wang , M. Xiong , C. Chen , et al., “Legumain, an Asparaginyl Endopeptidase, Mediates the Effect of M2 Macrophages on Attenuating Renal Interstitial Fibrosis in Obstructive Nephropathy,” Kidney International 94, no. 1 (2018): 91–101, 10.1016/j.kint.2017.12.025. 29656902

[25] X. Pei , S.‐L. Zhang , B.‐Q. Qiu , P.‐F. Zhang , T.‐S. Liu , and Y. Wang , “Cancer Cell Secreted Legumain Promotes Gastric Cancer Resistance to Anti‐PD‐1 Immunotherapy by Enhancing Macrophage M2 Polarization,” Pharmaceuticals 17, no. 7 (2024): 951, 10.3390/ph17070951. 39065799 PMC11279811

[26] S. G. Sun , J. J. Guo , X. Y. Qu , et al., “The Extracellular Vesicular Pseudogene LGMNP1 Induces M2‐Like Macrophage Polarization by Upregulating LGMN and Serves as a Novel Promising Predictive Biomarker for Ovarian Endometriosis Recurrence,” Human Reproduction 37, no. 3 (2022): 447–465, 10.1093/humrep/deab266. 34893848

[27] T. J. Standiford , “Macrophage Polarization in Sarcoidosis: An Unexpected Accomplice?,” American Journal of Respiratory Cell and Molecular Biology 60, no. 1 (2019): 9–10, 10.1165/rcmb.2018-0298ED. 30281325 PMC6835044

[28] S. Chen , A. F. U. H. Saeed , Q. Liu , et al., “Macrophages in Immunoregulation and Therapeutics,” Signal Transduction and Targeted Therapy 8, no. 1 (2023): 207, 10.1038/s41392-023-01452-1. 37211559 PMC10200802

[29] P. T. Elkington and J. S. Friedland , “Matrix Metalloproteinases in Destructive Pulmonary Pathology,” Thorax 61, no. 3 (2006): 259–266, 10.1136/thx.2005.051979. 16227332 PMC2080735

[30] A. O. Samokhin , J. Y. Gauthier , M. D. Percival , and D. Brömme , “Lack of Cathepsin Activities Alter or Prevent the Development of Lung Granulomas in a Mouse Model of Sarcoidosis,” Respiratory Research 12, no. 1 (2011): 13, 10.1186/1465-9921-12-13. 21251246 PMC3036631

[31] A. O. Samokhin , F. Bühling , F. Theissig , and D. Brömme , “ApoE‐Deficient Mice on Cholate‐Containing High‐Fat Diet Reveal a Pathology Similar to Lung Sarcoidosis,” The American Journal of Pathology 176, no. 3 (2010): 1148–1156, 10.2353/ajpath.2010.090857. 20093498 PMC2832138

[32] E. D. Crouser , D. A. Culver , K. S. Knox , et al., “Gene Expression Profiling Identifies MMP‐12 and ADAMDEC1 as Potential Pathogenic Mediators of Pulmonary Sarcoidosis,” American Journal of Respiratory and Critical Care Medicine 179, no. 10 (2009): 929–938, 10.1164/rccm.200803-490OC. 19218196 PMC2684019

[33] M. Vaalamo , A. L. Kariniemi , S. D. Shapiro , and U. Saarialho‐Kere , “Enhanced Expression of human Metalloelastase (MMP‐12) in Cutaneous Granulomas and Macrophage Migration,” Journal of Investigative Dermatology 112, no. 4 (1999): 499–505, 10.1046/j.1523-1747.1999.00547.x. 10201535

[34] A. A. González , A. M. Segura , K. Horiba , et al., “Matrix Metalloproteinases and Their Tissue Inhibitors in the Lesions of Cardiac and Pulmonary Sarcoidosis: An Immunohistochemical Study,” Human Pathology 33, no. 12 (2002): 1158–1164, 10.1053/hupa.2002.129423. 12514782

[35] T. Isshiki , H. Matsuyama , T. Yamaguchi , et al., “Plasma Matrix Metalloproteinase 7, CC‐Chemokine Ligand 18, and Periostin as Markers for Pulmonary Sarcoidosis,” Respiratory Investigation 58, no. 6 (2020): 479–487, 10.1016/j.resinv.2020.07.003. 32868264

[36] W. J. Piotrowski , P. Górski , T. Pietras , W. Fendler , and J. Szemraj , “The Selected Genetic Polymorphisms of Metalloproteinases MMP2, 7, 9 and MMP Inhibitor TIMP2 in Sarcoidosis,” Medical Science Monitor 17, no. 10 (2011): CR598–CR607, 10.12659/msm.881987. 21959615 PMC3539463

[37] P. Bai , L. Lyu , T. Yu , et al., “Macrophage‐Derived Legumain Promotes Pulmonary Hypertension by Activating the MMP (Matrix Metalloproteinase)‐2/TGF (Transforming Growth Factor)‐β1 Signaling,” Arteriosclerosis, Thrombosis, and Vascular Biology 39, no. 4 (2019): 130, 10.1161/atvbaha.118.312254. 30676070

[38] S. Freeley , J. Cardone , S. C. Günther , et al., “Asparaginyl Endopeptidase (Legumain) Supports Human Th1 Induction via Cathepsin L‐Mediated Intracellular C3 Activation,” Frontiers in Immunology 9 (2018): 2449, 10.3389/fimmu.2018.02449. 30405635 PMC6207624

[39] Y. He , P. Zou , J. Lu , et al., “CD4+ T‐Cell Legumain Deficiency Attenuates Hypertensive Damage via Preservation of TRAF6,” Circulation Research 134, no. 1 (2024): 9–29, 10.1161/circresaha.123.322835. 38047378

[40] C. Prior , R. A. Knight , M. Herold , G. Ott , and M. A. Spiteri , “Pulmonary Sarcoidosis: Patterns of Cytokine Release In Vitro,” European Respiratory Journal 9, no. 1 (1996): 47–53, 10.1183/09031936.96.09010047. 8834333

[41] G. Mangioris , S. J. Pittock , B. Yang , et al., “Cerebrospinal Fluid Cytokine and Chemokine Profiles in Central Nervous System Sarcoidosis: Diagnostic and Immunopathologic Insights,” Annals of Neurology 96, no. 4 (2024): 704–714, 10.1002/ana.27024. 39031103 PMC11568840

[42] T. TeSlaa , M. Ralser , J. Fan , and J. D. Rabinowitz , “The Pentose Phosphate Pathway in Health and Disease,” Nature Metabolism 5, no. 8 (2023): 1275–1289, 10.1038/s42255-023-00863-2. PMC11251397 37612403

[43] S. Prokop , F. L. Heppner , H. H. Goebel , and W. Stenzel , “M2 Polarized Macrophages and Giant Cells Contribute to Myofibrosis in Neuromuscular Sarcoidosis,” The American Journal of Pathology 178, no. 3 (2011): 1279–1286, 10.1016/j.ajpath.2010.11.065. 21356378 PMC3069905

[44] E. Dall and H. Brandstetter , “Mechanistic and Structural Studies on legumain Explain Its Zymogenicity, Distinct Activation Pathways, and Regulation,” Proceedings of the National Academy of Sciences 110, no. 27 (2013): 10940–10945, 10.1073/pnas.1300686110. PMC3703970 23776206

[45] Y. Zhang , C. Yan , Y. Dong , et al., “ANGPTL3 Accelerates Atherosclerotic Progression via Direct Regulation of M1 Macrophage Activation in Plaque,” Journal of Advanced Research 70 (2025): 125–138, 10.1016/j.jare.2024.05.011. 38740260 PMC11976407

[46] N. Shimojo , R. Hashizume , K. Kanayama , et al., “Tenascin‐C May Accelerate Cardiac Fibrosis by Activating Macrophages via the Integrin αVβ3/Nuclear Factor–κB/Interleukin‐6 Axis,” Hypertension 66, no. 4 (2015): 757–766, 10.1161/hypertensionaha.115.06004. 26238448

[47] K. M. Dhodapkar , D. Banerjee , J. Connolly , et al., “Selective Blockade of the Inhibitory Fcγ Receptor (FcγRIIB) in Human Dendritic Cells and Monocytes Induces a type I Interferon Response Program,” The Journal of Experimental Medicine 204, no. 6 (2007): 1359–1369, 10.1084/jem.20062545. 17502666 PMC2118610

[48] K. C. Goh , S. J. Haque , and B. R. Williams , “p38 MAP Kinase is Required for STAT1 Serine Phosphorylation and Transcriptional Activation Induced by Interferons,” The EMBO Journal 18, no. 20 (1999): 5601–5608, 10.1093/emboj/18.20.5601. 10523304 PMC1171628

[49] K. Ramsauer , I. Sadzak , A. Porras , et al., “p38 MAPK Enhances STAT1‐Dependent Transcription Independently of Ser‐727 Phosphorylation,” Proceedings of the National Academy of Sciences 99, no. 20 (2002): 12859–12864, 10.1073/pnas.192264999. PMC130550 12232043

[50] S. Uddin , A. Sassano , D. K. Deb , et al., “Protein Kinase C‐δ (PKC‐δ) Is Activated by Type I Interferons and Mediates Phosphorylation of Stat1 on Serine 727,” Journal of Biological Chemistry 277, no. 17 (2002): 14408–14416, 10.1074/jbc.M109671200. 11839738

[51] C. Rotenberg , V. Besnard , P.‐Y. Brillet , S. Giraudier , H. Nunes , and D. Valeyre , “Dramatic Response of Refractory Sarcoidosis Under Ruxolitinib in a Patient With Associated JAK2‐Mutated Polycythemia,” European Respiratory Journal 52, no. 6 (2018): 1801482, 10.1183/13993003.01482-2018. 30361243

[52] J. T. Rosenbaum , S. Pasadhika , E. D. Crouser , et al., “Hypothesis: Sarcoidosis Is a STAT1‐Mediated Disease,” Clinical Immunology 132, no. 2 (2009): 174–183, 10.1016/j.clim.2009.04.010. 19464956 PMC2733945

[53] W. Damsky , A. Wang , D. J. Kim , et al., “Inhibition of Type 1 Immunity With Tofacitinib Is Associated With Marked Improvement in Longstanding Sarcoidosis,” Nature Communications 13, no. 1 (2022): 3140, 10.1038/s41467-022-30615-x. PMC9170782 35668129

[54] W. Damsky , D. Thakral , M. K. McGeary , J. Leventhal , A. Galan , and B. King , “Janus Kinase Inhibition Induces Disease Remission in Cutaneous Sarcoidosis and Granuloma Annulare,” Journal of the American Academy of Dermatology 82, no. 3 (2020): 612–621, 10.1016/j.jaad.2019.05.098. 31185230 PMC7590533

[55] R. Kraaijvanger , C. A. Ambarus , J. Damen , et al., “Simultaneous Assessment of mTORC1, JAK/STAT, and NLRP3 Inflammasome Activation Pathways in Patients With Sarcoidosis,” International Journal of Molecular Sciences 24, no. 16 (2023): 12792, 10.3390/ijms241612792. 37628972 PMC10454122

[56] W. Damsky , D. Thakral , N. Emeagwali , A. Galan , and B. King , “Tofacitinib Treatment and Molecular Analysis of Cutaneous Sarcoidosis,” New England Journal of Medicine 379, no. 26 (2018): 2540–2546, 10.1056/NEJMoa1805958. 30586518 PMC6351852

[57] J. Talreja , C. Peng , and L. Samavati , “MIF Modulates p38/ERK Phosphorylation via MKP‐1 Induction in Sarcoidosis,” iScience 27, no. 1 (2024): 108746, 10.1016/j.isci.2023.108746. 38299032 PMC10829885

[58] R. Rastogi , W. Du , D. Ju , et al., “Dysregulation of p38 and MKP‐1 in Response to NOD1/TLR4 Stimulation in Sarcoid Bronchoalveolar Cells,” American Journal of Respiratory and Critical Care Medicine 183, no. 4 (2011): 500–510, 10.1164/rccm.201005-0792OC. 20851927 PMC5450927

[59] C. Zhang , Y. Wang , Z. Zhen , J. Li , J. Su , and C. Wu , “mTORC1 Mediates Biphasic Mechano‐Response to Orchestrate Adhesion‐Dependent Cell Growth and Anoikis Resistance,” Advanced Science 11, no. 6 (2024): 2307206, 10.1002/advs.202307206. 38041494 PMC10853740

[60] C. Chen , J. Gu , C. Yang , et al., “Targeting Integrin αVβ3–Ptgs2–mTOR Signaling Rescues Bone Formation in Osteoporosis: From Molecular Mechanism Toward Therapy,” Cellular & Molecular Biology Letters 31, no. 1 (2026): 17, 10.1186/s11658-025-00842-3. 41495647 PMC12870427

[61] L. Yang , N. Li , D. Yang , et al., “CCL2 Regulation of MST1‐mTOR‐STAT1 Signaling Axis Controls BCR Signaling and B‐cell Differentiation,” Cell Death & Differentiation 28, no. 9 (2021): 2616–2633, 10.1038/s41418-021-00775-2. 33879857 PMC8408168

[62] Y. Zhu , H. Gu , L. Yang , et al., “Involvement of MST1/mTORC1/STAT1 Activity in the Regulation of B‐Cell Receptor Signalling by Chemokine Receptor 2,” Clinical and Translational Medicine 12, no. 7 (2022): 887, 10.1002/ctm2.887. PMC9309749 35875970

[63] K. Ai , K. Li , X. Jiao , et al., “IL‐2–mTORC1 Signaling Coordinates the STAT1/T‐Bet Axis to Ensure Th1 Cell Differentiation and Anti‐Bacterial Immune Response in Fish,” PLOS Pathogens 18, no. 10 (2022): 1010913, 10.1371/journal.ppat.1010913. PMC9595569 36282845

[64] R. Gupta , M. A. Judson , and R. P. Baughman , “Management of Advanced Pulmonary Sarcoidosis,” American Journal of Respiratory and Critical Care Medicine 205, no. 5 (2022): 495–506, 10.1164/rccm.202106-1366CI. 34813386

[65] A. M. Shen and T. Minko , “Pharmacokinetics of Inhaled Nanotherapeutics for Pulmonary Delivery,” Journal of Controlled Release 326 (2020): 222–244, 10.1016/j.jconrel.2020.07.011. 32681948 PMC7501141

[66] E. D. Crouser , L. A. Maier , K. C. Wilson , et al., “Diagnosis and Detection of Sarcoidosis. An Official American Thoracic Society Clinical Practice Guideline,” American Journal of Respiratory and Critical Care Medicine 201, no. 8 (2020): e26–e51, 10.1164/rccm.202002-0251ST. 32293205 PMC7159433

[67] C. Huppertz , B. Jäger , G. Wieczorek , et al., “The NLRP3 Inflammasome Pathway Is Activated in Sarcoidosis and Involved in Granuloma Formation,” European Respiratory Journal 55, no. 3 (2020): 1900119, 10.1183/13993003.00119-2019. 31949113

[68] Y. Hu , Q. Wang , J. Yu , et al., “Tartrate‐Resistant Acid Phosphatase 5 Promotes Pulmonary Fibrosis by Modulating β‐catenin Signaling,” Nature Communications 13, no. 1 (2022): 114, 10.1038/s41467-021-27684-9. PMC8748833 35013220

[69] D. Chen , K. T. Love , Y. Chen , et al., “Rapid Discovery of Potent siRNA‐Containing Lipid Nanoparticles Enabled by Controlled Microfluidic Formulation,” Journal of the American Chemical Society 134, no. 16 (2012): 6948–6951, 10.1021/ja301621z. 22475086

[70] Q. Wang , J. Liu , Y. Hu , et al., “Local Administration of Liposomal‐Based Srpx2 Gene Therapy Reverses Pulmonary Fibrosis by Blockading Fibroblast‐to‐Myofibroblast Transition,” Theranostics 11, no. 14 (2021): 7110–7125, 10.7150/thno.61085. 34093874 PMC8171094

