# Supplementary material for: Legumain Restrains Granuloma Formation by Inhibiting mTORC1/STAT1‐Mediated M1 Macrophage Polarization in Sarcoidosis
Source: Adv Sci (Weinh). 2026 Apr 3;13(33):e20635. doi: 10.1002/advs.202520635 (PMC13271614; doi:10.1002/advs.202520635)
Supplement: Supplementary file 1 — Supporting File: advs75043‐sup‐0001‐SuppMat.pdf. [file ADVS-13-e20635-s001.pdf]

Supporting Information

Legumain Restrains Granuloma Formation by Inhibiting mTORC1/STAT1-Mediated M1 Macrophage Polarization in Sarcoidosis

Mengyuan Liu, Yueyin Han, Bingbing Xie, Lili Zhu, Wenxiu Xu, Yinzhen Han, Yue Liao, Shuwei Gao, Dingyuan Jiang, Jing Geng, Zhen Li, Yinan Hu*, Huaping Dai**

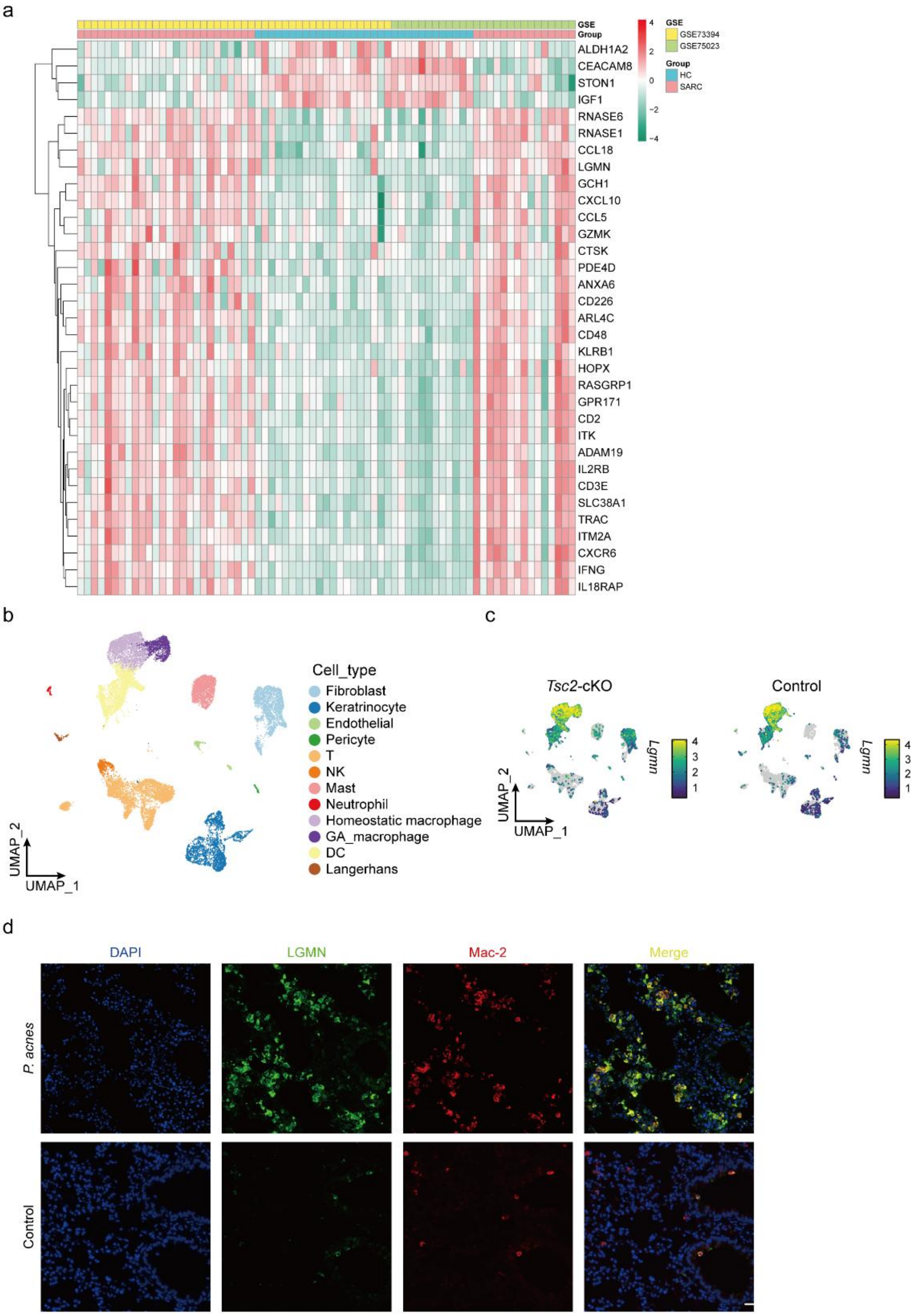

Figure S1. Validation of LGMN upregulation in sarcoidosis using transcriptomic datasets and mouse models. (a) Heatmap of shared DEGs, identified in two bulk RNA-seq datasets (GSE73394 and GSE75023), showing expression profiles in BAL cells between sarcoidosis patients and healthy donors. (b) UMAP of scRNA-seq transcriptome profiles from swollen paw and tail skin of female age-matched *Tsc2*$^{fl/fl}$ CD11c-Cre^{+} (*Tsc2*-cKO) sarcoid-like granuloma model mice and *Tsc2*$^{fl/fl}$ control littermate (n = 3 each). Transcriptomic data sources: GSE250508. (c) UMAP from (b) annotated with expression of *Lgmn*. (d) Representative images for co-immunostaining of LGMN (green) and Mac-2 (red) in the lung sections from control (n = 3) or *P. acnes*-induced mice (n = 5).

The nuclei were stained blue by DAPI. Images were captured at ×400 magnification. Bar = 20 μm.

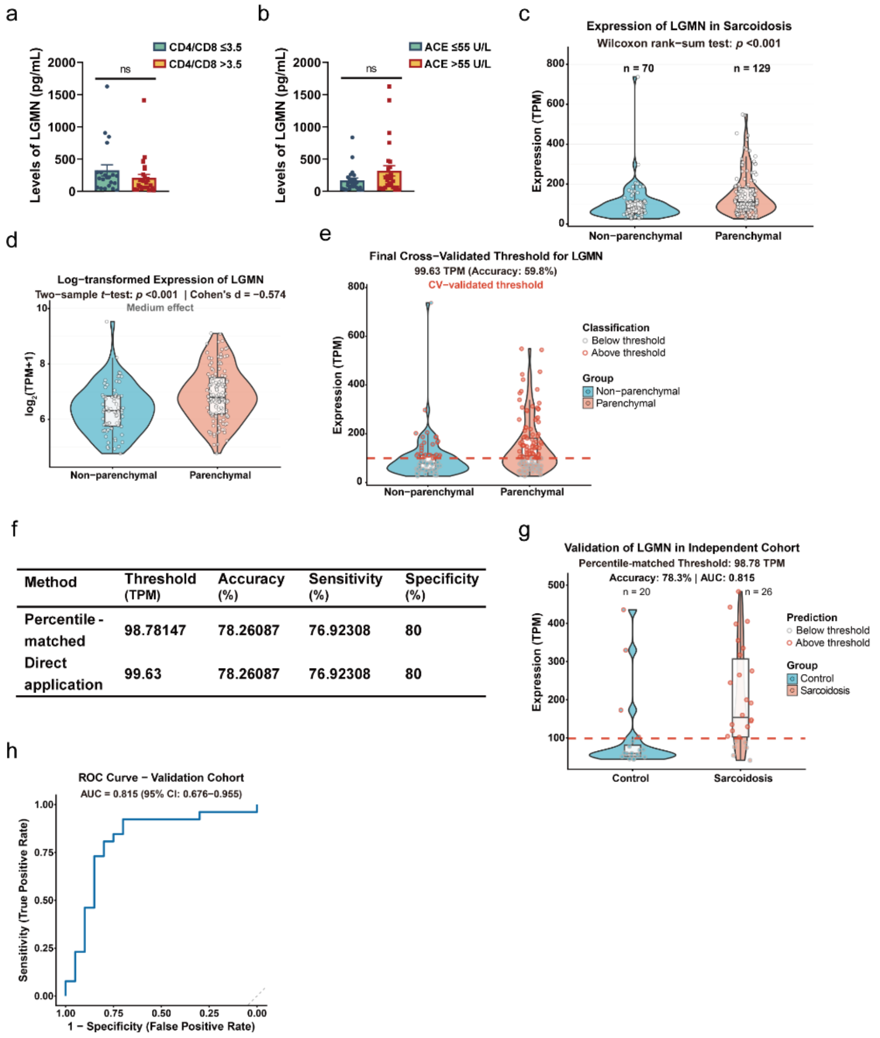

Method	Threshold (TPM)	Accuracy (%)	Sensitivity (%)	Specificity (%)
Percentile - matched	98.78147	78.26087	76.92308	80
Direct application	99.63	78.26087	76.92308	80

Figure S2. LGMN expression in BALF and its diagnostic performance for sarcoidosis. (a) ELISA analysis of LGMN levels in BALF from sarcoidosis patients stratified into those with a BALF CD4/CD8 ratio ≤3.5 (n = 21) and those with a ratio >3.5 (n = 27). Data are represented as the mean ± SEM. An unpaired *t*-test was applied. (b) ELISA analysis comparing BALF LGMN levels between sarcoidosis patients with low (≤55 U/L, n = 23) and high (>55 U/L, n = 27) serum ACE levels. The data are represented as the mean ± SEM. An unpaired *t*-test was applied. (c) Comparison of LGMN mRNA levels (TPM) in BAL cells between sarcoidosis patients with (n = 129) and without (n = 70) parenchymal involvement from the GSE109516 dataset. Data are shown as median with interquartile range. *P*-value was determined by the Wilcoxon rank-sum test. (d) Comparison of LGMN mRNA levels ($\log_2$[TPM+1]) in BAL cells between sarcoidosis patients with (n = 129) and without (n = 70) parenchymal involvement from the GSE109516 dataset. Data are shown as median with interquartile range. *P*-value was determined by the two-tailed Student's *t*-test. (e) Violin plot showing LGMN mRNA expression (TPM) in the internal validation cohort. The dashed line indicates the optimal cutoff value for LGMN. (f) Performance of the LGMN cutoff in the GSE73394 dataset using percentile matching and direct application methods. (g) Violin plot showing LGMN

mRNA expression (TPM) in the external validation cohort (GSE73394). (h) ROC curve validating the LGMN threshold in the external validation cohort (GSE73394).

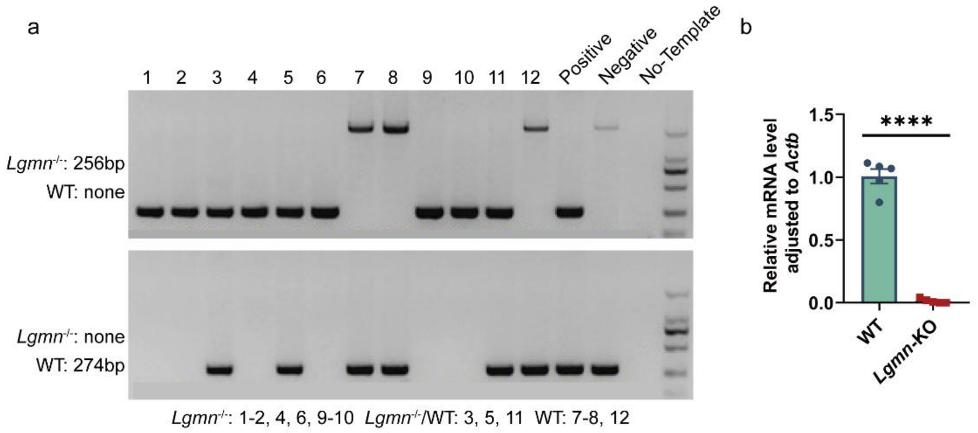

Figure S3. Validation of *Lgmn* knockout in mice. (a) Genotyping results of WT and *Lgmn*$^{-/-}$ allele. The wildtype allele is none, and *Lgmn*$^{-/-}$ allele is 256 bp (up). The wildtype allele is 274 bp, and the *Lgmn*$^{-/-}$ is none (below). (b) qRT-PCR analysis of the levels of *Lgmn* in the lungs from WT and *Lgmn*-KO mice following *P. acnes* challenge (n = 5 each). The data are represented as the mean ± SEM. An unpaired *t*-test was applied. * $p < 0.05$, ** $p < 0.01$, *** $p < 0.001$, **** $p < 0.0001$.

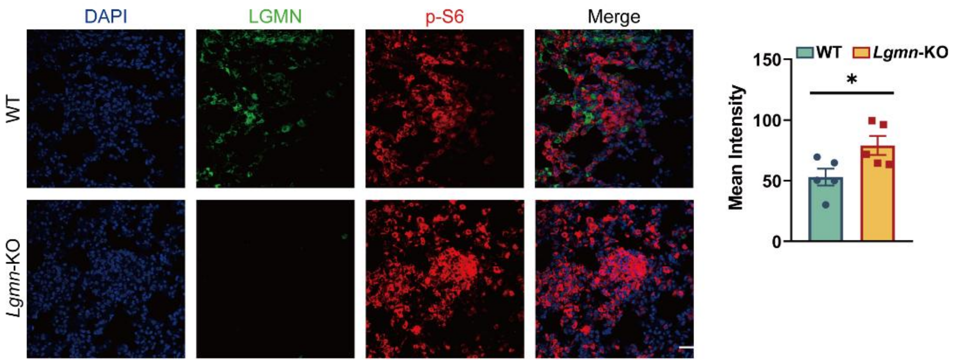

Figure S4. Co-immunostaining of LGMN and p-S6. Co-immunostaining of LGMN (green) and p-S6 (red) in lung sections from WT and *Lgmn*-KO mice after *P. acnes* induction, with quantification of p-S6 mean fluorescence intensity (n = 5 each). The nuclei were stained blue by DAPI. Images were captured at ×400 magnification. Bar = 25 μm. The data are represented as the mean ± SEM. An unpaired *t*-test was applied. * $p < 0.05$, ** $p < 0.01$, *** $p < 0.001$, **** $p < 0.0001$.

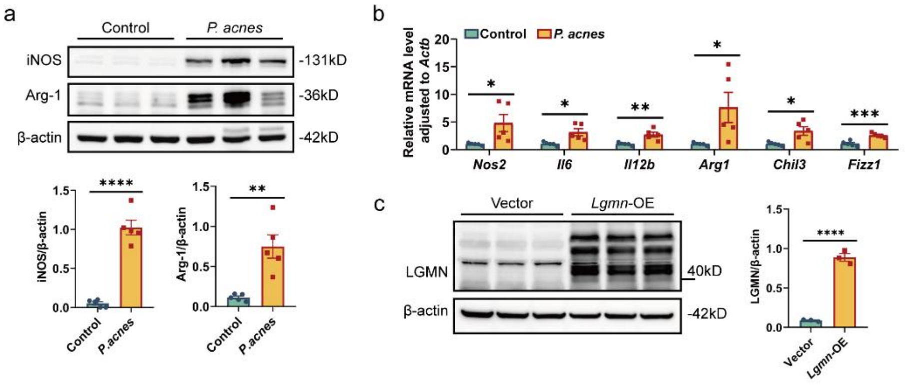

Figure S5. Analysis of macrophage polarization in sarcoid-like granulomas and validation of *Lgmn* overexpression in transfected HEK-293T cells. (a) Western blot analysis for expression of iNOS and Arg-1 in the lungs of mice with or without *P. acnes* induction (n = 5 each). (b) qRT-PCR analysis of *Nos2, Il6, Il12b, Arg1, Chil3,* and *Fizz1* expression in the lungs from PBS and *P. acnes*-induced mouse model (n = 5 each). (c) Western blot analysis for LGMN expression in vector control or *Lgmn* palsmid-treated HEK-293T cells. The data are represented as the mean ± SEM. An unpaired *t*-test was applied. * $p < 0.05$, ** $p < 0.01$, *** $p < 0.001$, **** $p < 0.0001$.

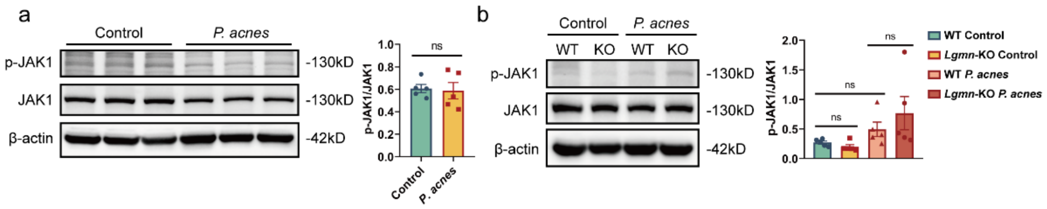

Figure S6. The effects of LGMN on JAK1 activation. (a) Western blot analysis of the levels of p-JAK1 and JAK1 in the lungs from mice with or without *P. acnes* challenge (n = 5 each). (b) Western blot analysis of p-JAK1 and JAK1 expression in the lungs of WT and *Lgmn*-KO mice after *P. acnes* induction (n = 5 each). The data are represented as the mean ± SEM. An unpaired *t*-test (a) and ordinary one-way ANOVA (b) were applied. * $p < 0.05$, ** $p < 0.01$, *** $p < 0.001$, **** $p < 0.0001$.

a

	Lgmn-pDNA loaded LNPs
Hydrodynamic diameter (nm)	94.11
PDI	0.216
Zeta-potential (mv)	-2.78
Entrapment efficiency (%)	69.15

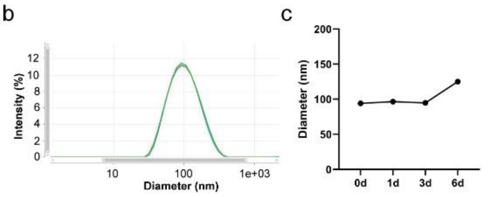

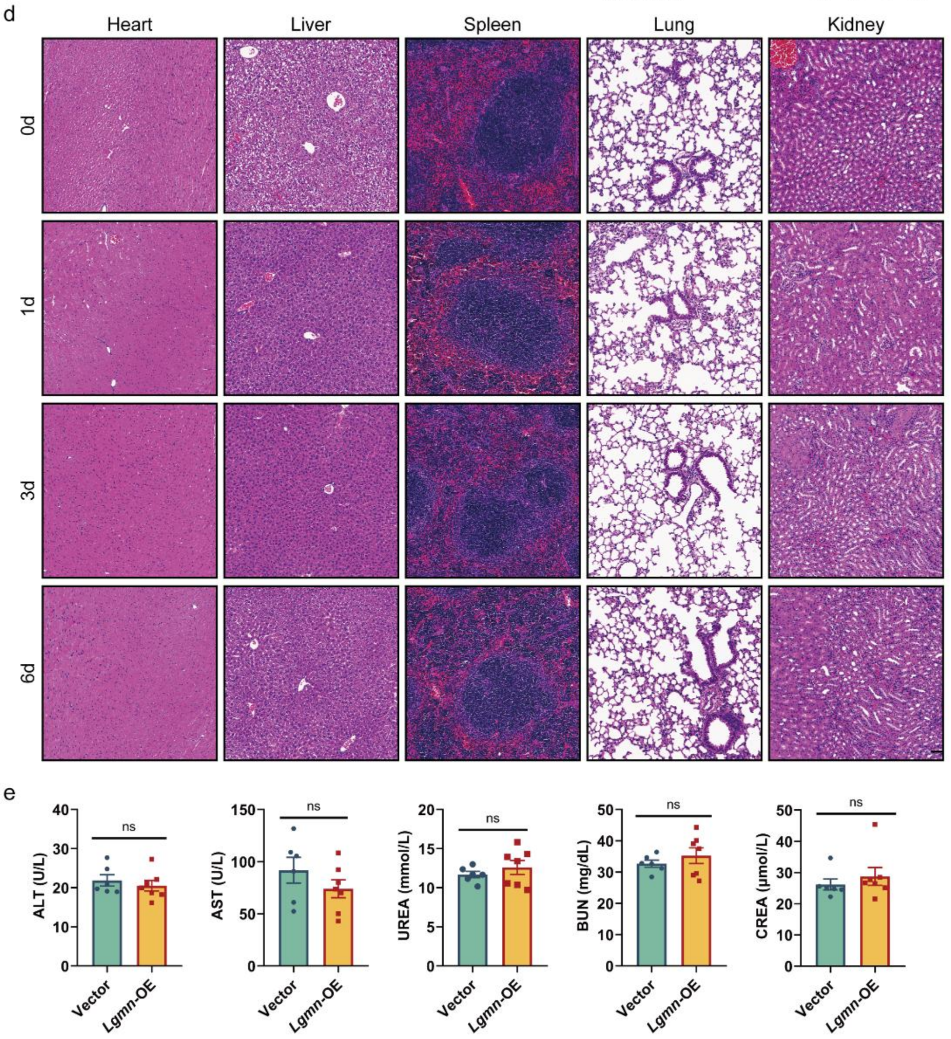

Figure S7. The characterization and biosafety of *Lgmn* pDNA-loaded LNPs. (a) The average size, polydispersity index (PDI), zeta potential, and entrapment efficiency of *Lgmn* pDNA-loaded LNPs. (b) The distribution of hydrodynamic diameter of those LNPs carrying *Lgmn* pDNA within 24 hours (n = 3). (c) The change of hydrodynamic diameter of the prepared LNPs at different timepoints (n = 3). (d) H&E staining of heart, liver, spleen, lung, and kidney in mice (n = 3 each) after treatment with *Lgmn* pDNA-loaded LNPs. Images were captured at ×200 magnification. Bar = 50 μm. (e) Serum biochemical analysis of mice treated with vector-loaded LNPs (n = 6) or *Lgmn* pDNA-loaded LNPs (n = 7). The data are represented as the mean ± SEM. An unpaired *t*-test was applied.

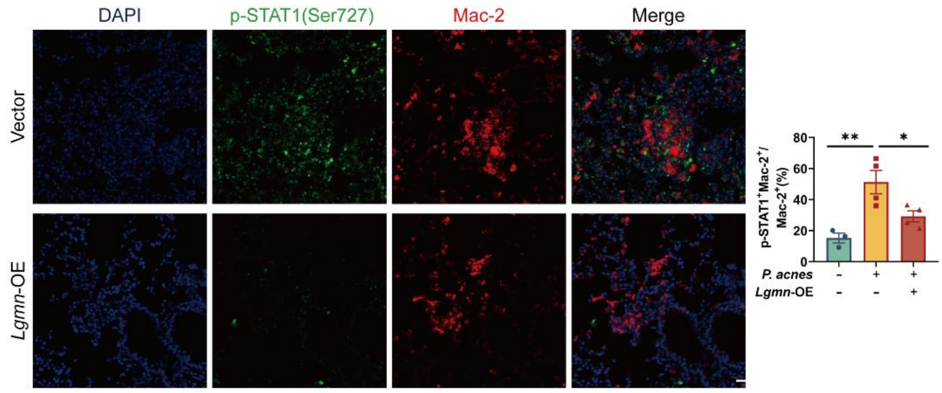

Figure S8. Co-immunostaining of p-STAT1(Ser727) and Mac-2. Results for co-immunostaining of p-STAT1(Ser727) (green) and Mac-2 (red) in the lung sections from Control group (n = 3), Vector group (n = 4), and *Lgmn*-OE group (n = 4). The nuclei were stained blue by DAPI. Images were captured at ×400 magnification. Bar = 20 μm. The data are represented as the mean ± SEM. Ordinary one-way ANOVA was applied. * $p < 0.05$, ** $p < 0.01$, *** $p < 0.001$, **** $p < 0.0001$.

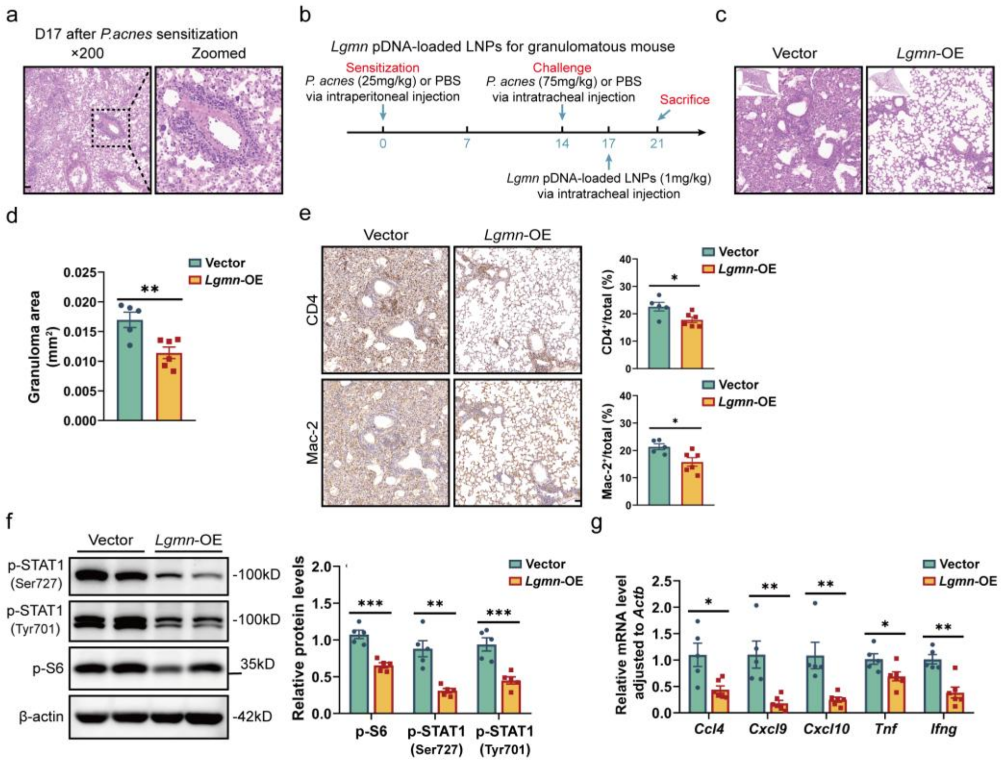

Figure S9. Therapeutic efficacy of *Lgmn* pDNA-loaded LNPs against established *P. acnes*-induced granulomas. (a) Representative images of H&E-stained lung sections from the mice 17 days after *P. acnes* sensitization. Images were captured at ×200 magnification. Bar = 50 µm. (b) Schematic of *Lgmn* pDNA-loaded LNPs treatment design. (c) Representative images of H&E staining of lung sections from the mice described in (b). Bar = 50 µm. (d) Mean granuloma area in mice treated with vector-loaded LNPs (n = 5) or *Lgmn* pDNA-loaded LNPs (n = 6) on day 21 after *P. acnes* sensitization. (e) IHC staining and quantification for CD4 and Mac-2 in lung sections from *P. acnes*-induced mice treated with vector- (n = 5) or *Lgmn* pDNA-loaded LNPs (n = 6). Representative images were captured at ×200 magnification. Bar = 50 µm. (f) Western blot analysis of p-STAT1 (Ser727 and Tyr701) and p-S6 expression in lung tissues from mice treated with vector-loaded LNPs or *Lgmn* pDNA-loaded LNPs (n = 5 per group). (g) qRT-PCR analysis of *Ccl4, Cxcl9, Cxcl10, Tnf,* and *Ifng* expression in lung homogenates from mice treated with vector-loaded LNPs (n = 5) and *Lgmn* pDNA-loaded LNPs (n = 6). The data are represented as the mean ± SEM. An unpaired *t*-test was applied. * $p < 0.05$, ** $p < 0.01$, *** $p < 0.001$, **** $p < 0.0001$.

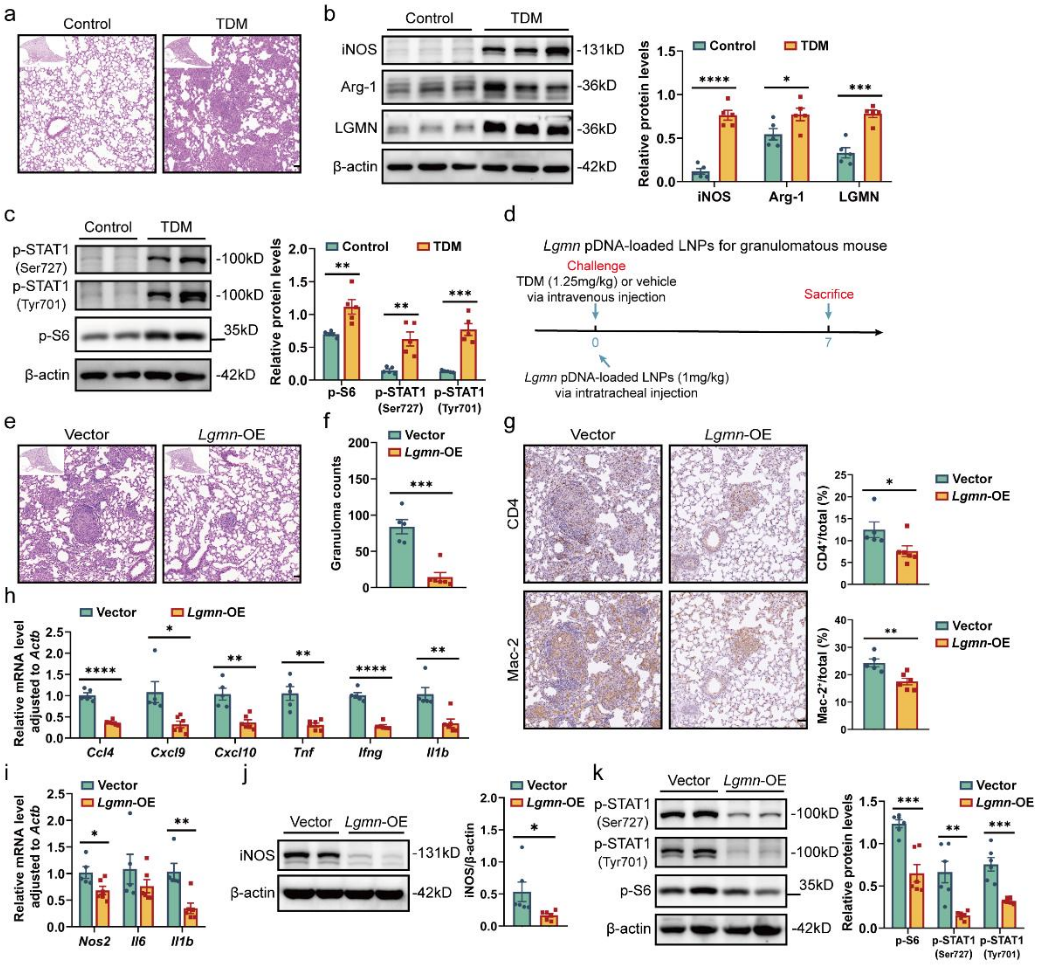

Figure S10. Treatment with LNPs carrying *Lgmn* pDNA ameliorates TDM-induced pulmonary granulomatous inflammation. (a) H&E staining of lung sections harvested on day 7 from mice challenged with TDM or vehicle (Control). Representative images were captured at ×200 magnification. Bar = 50 μm. (b) Western blot analysis of iNOS, Arg-1, and LGMN expression in the lungs of vehicle- or TDM-administered mice (n = 5 each). (c) Western blot analysis of the levels of p-STAT1(Ser727 and Tyr701) and p-S6 in lung tissues from control or TDM-induced mice (n = 5 each). (d) Schematic diagram of *Lgmn* pDNA-loaded LNPs treatment in TDM-challenged mice. (e) Representative images of H&E staining of lung sections from the mice treated with vector-loaded LNPs (Vector) or *Lgmn* pDNA-loaded LNPs (*Lgmn*-OE), shown at ×200 magnification. Bar = 50 μm. (f) Granuloma counts in mice treated with vector- (n = 5) or *Lgmn* pDNA-loaded LNPs (n = 6) on day 7 after TDM injection. (g) IHC staining and quantification for CD4 and Mac-2 in lung sections from TDM-injected mice treated with vector-loaded LNPs (n = 5) or *Lgmn* pDNA-loaded LNPs (n = 6). Representative images were captured at ×200 magnification. Bar = 50 μm. (h) qRT-PCR analysis of *Ccl4, Cxcl9, Cxcl10, Tnf, Ifng,* and *Il1b* expression in lung tissues from mice treated with vector-loaded LNPs (n = 5) or *Lgmn* pDNA-loaded LNPs (n = 6). (i) qRT-PCR analysis of M1 polarization-related markers (*Nos2, Il6,* and *Il1b*) in the lung homogenates from mice with or without *Lgmn* pDNA-loaded LNPs treatment (n = 5-6 each). (j) Western blot analysis of iNOS expression in the lungs of vector- and *Lgmn* pDNA-loaded

LNPs-treated mice (n = 6 each). (k) Western blot analysis of p-STAT1(Ser727 and Tyr701) and p-S6 expression in the lungs of vector- and *Lgmn* pDNA-loaded LNPs-treated mice (n = 6 each). The data are represented as the mean ± SEM. An unpaired *t*-test was applied. * $p < 0.05$, ** $p < 0.01$, *** $p < 0.001$, **** $p < 0.0001$.

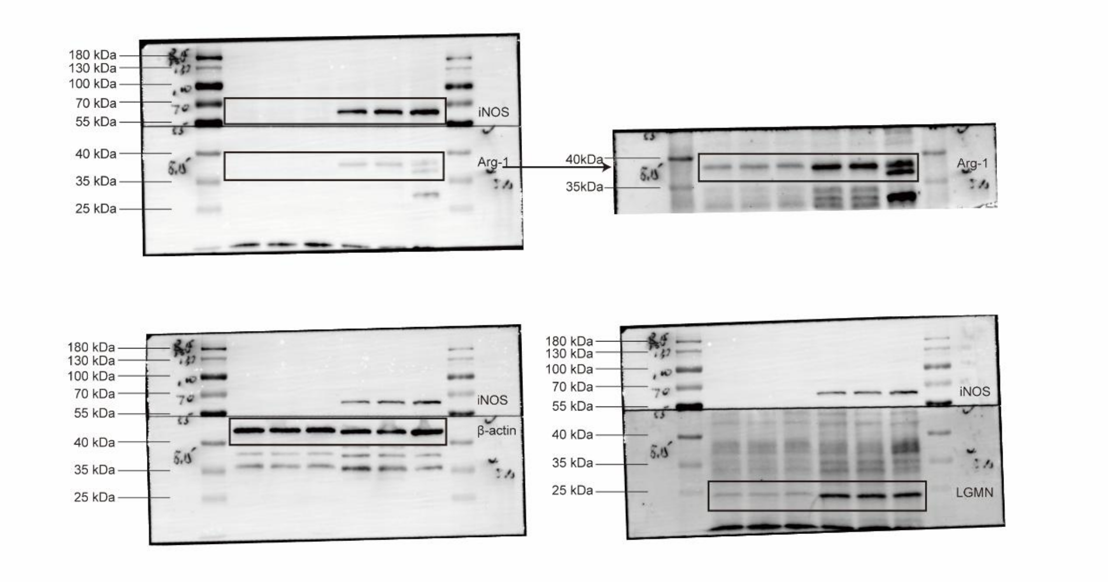

Figure S11. Raw, unedited scans of Western blot membranes corresponding to Figure 1b and 3a. Membranes were sectioned according to the molecular weights of the target proteins before antibody incubation. The sections were subsequently reassembled for imaging. All bands shown in Figure 1b and 3a were derived from the same membrane.

Table S1. Characteristics of the Patients

Count	Diagnosis	Gender	Age	Tissue Type
1	Sarcoidosis	Female	39	FFPE lymph node section
2	Sarcoidosis	Female	56	FFPE lymph node section
3	Sarcoidosis	Female	52	FFPE lymph node section
4	Sarcoidosis	Female	61	FFPE lymph node section
5	Sarcoidosis	Male	57	FFPE lymph node section
6	Sarcoidosis	Female	54	FFPE lung section
7	Sarcoidosis	Female	58	FFPE lung section
8	Sarcoidosis	Female	57	FFPE lung section
9	Sarcoidosis	Female	41	FFPE lung section
10	Sarcoidosis	Male	54	FFPE lung section
11	Sarcoidosis	Female	58	Fresh-frozen lymph nodes
12	Sarcoidosis	Female	64	Fresh-frozen lymph nodes
13	Sarcoidosis	Male	61	Fresh-frozen lymph nodes

Table S2. Primer sequence used in the present study.

Target	Forward	Reverse
Lgmn	ATCAACCGACCTAACGGCACAG	ACAGCTTCTGCGTCACCTCTCA
Ccl2	GCTACAAGAGGATCACCAGCAG	GTCTGGACCCATTCCTTCTTGG
Ccl3	ACTGCCTGCTGCTTCTCCTACA	ATGACACCTGGCTGGGAGCAAA
Ccl4	ACCCTCCCACTTCCTGCTGTTT	CTGTCTGCCTCTTTTGGTCAGG
Cxcl9	CCTAGTGATAAGGAATGCACGATG	CTAGGCAGGTTTGATCTCCGTTC
Cxcl10	ATCATCCCTGCGAGCCTATCCT	GACCTTTTTTGGCTAAACGCTTTC
Tnf	GGTGCCTATGTCTCAGCCTCTT	GCCATAGAACTGATGAGAGGGAG
Ifng	CAGCAACAGCAAGGCGAAAAAGG	TTTCCGCTTCCTGAGGCTGGAT
Il17a	CAGACTACCTCAACCGTTCCAC	TCCAGCTTTCCCTCCGCATTGA
Il6	TACCACTTCACAAGTCGGAGGC	CTGCAAGTGCATCATCGTTGTTC
Il1b	TGGACCTTCCAGGATGAGGACA	GTTCATCTCGGAGCCTGTAGTG
Il12b	TTGAACTGGCGTTGGAAGCACG	CCACCTGTGAGTTCTTCAAAGGC
Nos2	GAGACAGGGAAGTCTGAAGCAC	CCAGCAGTAGTTGCTCCTCTTC
Arg1	CATTGGCTTGCGAGACGTAGAC	GCTGAAGGTCTCTTCCATCACC
Chil3	TACTCACTTCCACAGGAGCAGG	CTCCAGTGTAGCCATCCTTAGG
Fizz1	CAAGGAACTTCTTGCCAATCCAG	CCAAGATCCACAGGCAAAGCCA
Hk2	CCCTGTGAAGATGTTGCCCACT	CCTTCGCTTGCCATTACGCACG
Slc2a1	GCTTCTCCAACTGGACCTCAAAC	ACGAGGAGCACCGTGAAGATGA
Pfkfb3	TCATCGAGTCGGTCTGTGACGA	CATGGCTTCTGCTGAGTTGCAG
Pkm	CAGAGAAGGTCTTCCTGGCTCA	GCCACATCACTGCCTTCAGCAC
Ldha	ACGCAGACAAGGAGCAGTGGAA	ATGCTCTCAGCCAAGTCTGCCA
Actb	CATTGCTGACAGGATGCAGAAGG	TGCTGGAAGGTGGACAGTGAGG

Table S3. Urinalysis parameters in mice treated with vector- or *Lgmn* pDNA-loaded LNPs.

Parameter	Vector (n = 6)	*Lgmn*-OE (n = 7)	*P*-value[a]
WBC			
Negative	6 (100.0%)	7 (100.0%)	1.000
Positive	0 (0.0%)	0 (0.0%)	
KET			
Negative	6 (100.0%)	7 (100.0%)	1.000
Positive	0 (0.0%)	0 (0.0%)	
URO, μmol/L			
< 33	6 (100.0%)	7 (100.0%)	1.000
⩾33	0 (0.0%)	0 (0.0%)	
BIL			
Negative	6 (100.0%)	7 (100.0%)	1.000
Positive	0 (0.0%)	0 (0.0%)	
PRO, g/L			
0.15	6 (100.0%)	6 (85.7%)	>0.999
0.3	0 (0.0%)	1 (14.3%)	
GLU			
Negative	6 (100.0%)	7 (100.0%)	1.000
Positive	0 (0.0%)	0 (0.0%)	
SG			
1.020	0 (0.0%)	4 (57.1%)	0.070
1.025	5 (83.3%)	3 (42.9%)	
1.030	1 (16.7%)	0 (0.0%)	
BLD			
Negative	6 (100.0%)	7 (100.0%)	1.000
Positive	0 (0.0%)	0 (0.0%)	
PH			
5.0	1 (16.7%)	0 (0.0%)	0.233
5.5	4 (66.7%)	3 (42.9%)	
6.0	1 (16.7%)	0 (0.0%)	
6.5	0 (0.0%)	2 (28.6%)	
7.0	0 (0.0%)	2 (28.6%)	
CR, mmol/L			
⩽0.9	6 (100.0%)	6 (85.7%)	>0.999
4.4	0 (0.0%)	1 (14.3%)	
Ca, mmol/L			
2.5	1 (16.7%)	0 (0.0%)	0.462
5.0	5 (83.3%)	7 (100.0%)	
MA, mg/L			
<100	0	0	1.000
⩾100	6 (100.0%)	7 (100.0%)	

Data are presented as the number of animals (percentage within group).

[a]All *P*-values were calculated using Fisher's exact test (Freeman-Halton extension for tables larger than 2×2).
